# A novel q-ROHFS prospect theory based MABAC method for failure mode risk prioritization in aircraft landing systems

**DOI:** 10.1038/s41598-025-26585-x

**Published:** 2025-11-27

**Authors:** Ali Köseoğlu, Fatma Altun, Rıdvan Şahin

**Affiliations:** 1 Department of Mathematics, Recep Tayyip Erdogan University, Rize, Turkey; 2 Vocational School of Computer Technology, Gumushane University, Gümüşhane, Turkey; 3 Department of Software Engineering, Gumushane University, Gümüşhane, Turkey

**Keywords:** Q-rung orthopair hesitant fuzzy sets, FMEA, Prospect theory, MABAC, Best-worst method, Aircraft landing systems, Engineering, Mathematics and computing

## Abstract

Failure mode risk prioritization is crucial in aircraft landing systems, where undetected or misjudged failures can lead to catastrophic outcomes. Effective risk analysis enables proactive maintenance and enhances aviation safety in such critical phases of flight. In this study, a novel hybrid decision-making framework is proposed to prioritize failure modes in aircraft landing systems by integrating the Multi-Attributive Border Approximation Area Comparison (MABAC) method with Prospect Theory under a q-Rung Orthopair Hesitant Fuzzy Set (q-ROHFS) environment. Traditional failure modes and effects analysis (FMEA) approaches often suffer from rigid weighting schemes, lack of sensitivity to expert hesitancy, and an inability to incorporate psychological factors such as risk aversion or subjective evaluations—especially in high-risk domains like aviation. To address these limitations, the proposed model incorporates human psychological behaviour and uncertainty in expert assessments. Prospect Theory is employed to capture decision makers’ risk attitudes and reference-dependent evaluations, while q-ROHFSs allow more flexible and comprehensive representation of hesitant and uncertain information. In this approach, Best-Worst Method (BWM) is used to determine the relative importance of risk factors for each decision maker, and their individual weights are obtained using TOPSIS-based similarity measures. A novel generalized q-ROHF Minkowski distance measure is also introduced and implemented to determine the weights of decision makers in the TOPSIS method, as well as to construct the prospect decision matrix and the distance matrix in the MABAC method, thereby enhancing computational precision. The applicability and effectiveness of the proposed method are demonstrated through a real-world case study on aircraft landing systems, and a sensitivity analysis is conducted to validate the robustness of the results. The findings highlight the method’s capability to reflect expert preferences more realistically and improve risk prioritization decisions in complex safety-critical systems.

## Introduction

In safety-critical sectors such as aviation, identifying and eliminating risks before failures occur is of vital importance for ensuring operational continuity and protecting human lives. Among the various phases of flight, takeoff and landing are considered the most risk-prone and technically demanding operations, accounting for a significant portion of aviation accidents. In recent years, with the substantial increase in air traffic, especially at major airports, aircraft takeoffs and landings occur as frequently as every five minutes. In large-scale hubs such as the newly built Istanbul Airport, simultaneous takeoffs and landings of up to three aircraft are operationally possible. Considering the high number of passengers transported daily, a thorough analysis of risk factors has become critically important. According to the European Union Aviation Safety Agency (EASA) and the United States Federal Aviation Administration (FAA) safety statistics^1,2^ the majority of fatal accidents occur during the final approach and landing phases, primarily due to failures in the landing gear systems, brake mechanisms, hydraulic systems, sensor malfunctions, or control system faults. Detailed data on each incident and accident are available in both written and audio formats, providing valuable insights into failure patterns and risk propagation. Modern aircraft landing systems are highly integrated mechatronic assemblies that consist of mechanical components (e.g., landing gear, actuators), electronic systems (e.g., sensors, proximity switches), and software-based decision support mechanisms. The complex interactions among these subsystems significantly complicate the tasks of fault prediction and risk prioritization. Even a minor undetected or misclassified fault can lead to serious and potentially catastrophic outcomes such as runway excursions, gear collapse, tire bursts, or unstable approaches. In some cases, even microscopic wear on engine blades may result in flight cancellations. Therefore, risk assessment in this domain requires precise, systematic, and adaptive methods that can incorporate expert knowledge, technical uncertainty, and cognitive biases of decision-makers.

The idea of assessing risks in an event and acting accordingly dates back to the 1940s. During this period, the United States military pioneered the development of Failure Mode and Effects Analysis (FMEA), aiming to evaluate and prioritize potential risks in critical military operations. This method later found application in other high-stakes fields such as the NASA Apollo program and nuclear energy systems. As a result, FMEA has long been recognized as a widely accepted technique in the field of risk assessment. It provides a structured framework to evaluate possible failure modes based on factors such as the severity of their consequences, the likelihood of occurrence, and their detectability. However, conventional FMEA approaches typically rely on crisp numerical values, often assuming equal weighting of factors and consistency across expert opinions, which can be overly simplistic for complex aviation applications. Moreover, these methods fail to account for the psychological behaviour of decision-makers and the inherent uncertainty and hesitation in their judgments.

To better address such uncertainties and vagueness in expert evaluations, fuzzy set theory introduced by Zadeh^3^ has been widely employed in risk assessment models. Unlike traditional binary logic, fuzzy sets allow decision-makers to express their judgments with degrees of membership, thus offering a more nuanced representation of real-world imprecision. Later, intuitionistic fuzzy sets^4^ extended this framework by incorporating both membership and non-membership degrees, capturing hesitation more explicitly. These models are particularly valuable when expert knowledge is incomplete, conflicting, or subjective—as is often the case in aviation safety evaluations. By utilizing these fuzzy logic-based frameworks, decision support systems can better reflect human reasoning and ambiguity in complex environments. Therefore, the integration of fuzzy and intuitionistic fuzzy theories into FMEA-based methodologies significantly enhances the robustness and credibility of risk prioritization outcomes in aviation systems.

While fuzzy and intuitionistic fuzzy sets provide an essential basis for modelling uncertainty, they are often insufficient to fully represent hesitancy and complex cognitive ambiguity in expert evaluations. To overcome these limitations, hesitant fuzzy sets^5^ were introduced, allowing multiple membership values to express hesitation more explicitly. Building upon this, Yager^6^ proposed the q-rung orthopair fuzzy sets, which further generalize intuitionistic and Pythagorean fuzzy frameworks by relaxing the constraint on the sum of powered membership and non-membership degrees. This generalization provides greater flexibility in modelling uncertainty, particularly in high-risk environments like aviation systems.

Later, Liu et al.^7^ extended this concept to define q-rung orthopair hesitant fuzzy sets (q-ROHFS), combining the advantages of both hesitant and orthopair structures. In q-ROHFS, each element is characterized by a set of possible membership and non-membership values, with the q-th powered sum of their maximum degrees not exceeding one. This structure enables the representation of multiple concurrent expert opinions, varying confidence levels, and differing degrees of uncertainty within a unified mathematical framework.

In parallel with the development of fuzzy logic-based models, multi-criteria decision-making (MCDM) methods have played a significant role in improving risk evaluation processes under uncertainty. Alongside studies that conduct multi-criteria decision-making without using any MCDM^8^, classical MCDM methods such as AHP^9^, TOPSIS^10^, VIKOR^11^, CODAS^12^ and PROMETHEE^13^ have been extensively applied in engineering, medical, logistics, and safety-critical domains. These methods help rank or select alternatives by considering multiple conflicting criteria. More recently, advanced MCDM techniques—such as the Multi-Attributive Border Approximation Area Comparison (MABAC) method^14^—have gained attention due to their simplicity, computational efficiency, and robustness in handling complex decision matrices.

In the context of aviation systems, MCDM models are particularly useful in structuring risk evaluation problems where several interdependent factors (e.g., severity, likelihood, detectability) must be considered simultaneously. Furthermore, integrating MCDM methods with fuzzy set extensions allows for more flexible and realistic modelling of expert opinions. When these methods are further enriched with behavioural theories, such as Prospect Theory^15^, they can capture not only the quantitative uncertainty but also the qualitative psychological tendencies of decision-makers.

In this study, q-ROHFS is utilized to model expert evaluations of failure modes in aircraft landing systems, where uncertainty, subjectivity, and psychological factors are pronounced. Its integration into the FMEA-MABAC methodology allows for more realistic, human-aligned, and data-rich risk prioritization, setting the foundation for a more robust and comprehensive decision-making process. The rest of the paper is organized as below:

“Literature review, gap and motivation” presents a literature review on FMEA applications in aviation, identifies the existing research gaps, and outlines the motivation and objectives of this study. “Preliminaries” introduces the fundamental concepts and defines a new metric measure for q-Rung Orthopair Hesitant Fuzzy Sets (q-ROHFSs). In “Proposed FMEA model”, the methodologies used to determine the weights of decision makers and criteria are described, and the proposed extended FMEA model integrating MABAC with prospect theory is detailed. “A case study at risk assessment problem of aircraft landing” applies the proposed approach to a risk assessment problem in aircraft landing systems, including a sensitivity analysis and a comprehensive comparative evaluation. Finally, “Conclusion and future study” summarizes the study, highlights key conclusions, and discusses limitations and directions for future research.

## Literature review, gap and motivation

### q-Rung orthopair hesitant fuzzy sets and MABAC method

q-ROHFS is a recent and flexible extension of fuzzy set theory, introduced by Liu et al.^7^, to handle uncertainty and hesitation in expert evaluations more comprehensively. It generalizes Intuitionistic fuzzy sets by allowing the sum of the q-th powers of membership and non-membership degrees to be at most one. Additionally, the hesitant feature enables experts to express multiple possible membership values instead of a single crisp value, providing richer semantic representation. These sets have gained significant attention in recent years due to their enhanced modelling capabilities. For instance, Ghoushchi et al.^16^ utilized q-ROHFSs to evaluate uncertainty in subsidence cause factors. Hassan et al.^17^ proposed novel aggregation operators and similarity measures for q-ROHFSs. Xin et al.^18^ integrated q-ROHFSs with the VIKOR method, while Wan et al.^19^ employed q-ROHF preference relations in group decision making. The MABAC method, introduced by Pamucar and Cirovic^14^, is a widely applied MCDM technique known for its simplicity, computational efficiency, and clear distance-based ranking mechanism. It evaluates alternatives based on their distances to an ideal border approximation area. Over time, several extensions of the MABAC method have been developed using different types of fuzzy and uncertain sets. Fan et al.^20^ applied the MABAC method under cumulative prospect theory to evaluate wearable devices. Debnath et al.^21^ extended MABAC method with aggregation operators on q-ROFSs. Jafari and Khanachah^22^ employed the MABAC method for evaluation of suppliers. Ali and Yang^23^ also proposed a novel decision-making approach based on the MABAC method.

Although q-ROFS and the MABAC method have been extensively used individually in the literature, there also exist several studies that combine these two frameworks in integrated decision-making models lately. Naz et al.^24^ combined q-ROF information with MABAC method to solve a risk investment problem. Ali et al.^25^ utilized circular q-ROFS within a MABAC-based framework for applications in tissue engineering and regenerative medicine. Soni et al.^26^ developed a combined method to select recycled waste plastics.

Despite these developments, the integration of Prospect Theory with q-ROHFS-based MABAC frameworks remains largely underexplored in the current literature. Prospect Theory has been widely adopted in behavioural economics and decision-making under risk since it provides a valuable tool to model reference-dependent preferences and loss–gain asymmetry in expert judgments. Some recent studies have incorporated it into MCDM methods such as TOPSIS^27^ or TODIM^28^; however, its collaboration with advanced fuzzy environments, particularly q-ROHFSs, and distance-based MCDM techniques like MABAC has not been adequately addressed. This gap is especially critical for safety-critical domains such as aviation risk assessment, where both human behavioural biases and high degrees of uncertainty must be considered simultaneously. Therefore, this study aims to fill this important research gap by proposing a unified decision-making model that combines q-ROHFS, Prospect Theory, and MABAC in an integrated FMEA framework.

### FMEA applications in the aviation sector

FMEA has long been a key tool for systematically identifying, evaluating, and mitigating risks in aviation systems. Its structured framework for prioritizing potential failure modes based on severity, occurrence, and detectability has made it widely applicable in critical aviation subsystems, particularly under the constraints of safety, reliability, and real-time performance requirements. In the aviation domain, risk assessment has been applied in various contexts lately, such as aircraft brake systems^29–31^, aircraft engine components^32,33^, avionics systems^34–36^, and even unmanned aerial vehicle (UAV) reliability assessments^37,38^. In addition to these studies, FMEA and MCDM are used together to better represent the decisions in expert evaluations^39–43^ .

However, many of these approaches still rely on conventional techniques or basic fuzzy sets, and few address critical flight phases such as landing, where failure consequences are most severe. Additionally, despite its long history and widespread adoption, the traditional FMEA method has several well-known limitations, particularly when applied to high-risk, complex systems such as aircraft landing mechanisms such as equal weighting assumption, dependence of crisp judgements and lack of ranking robustness^44^. In particular, studies mentioned above mostly focus on aircraft landing gear systems and related components (e.g., hydraulic actuators, sensors, proximity switches), however, the psychological dimension of expert evaluation—such as hesitation, inconsistency, and risk perception—is often overlooked. Although these are recent works in aerospace maintenance and safety analysis, the absence of advanced fuzzy frameworks (e.g., q-ROHFS) and behavioural decision models in current aviation-focused FMEA studies highlights an important methodological gap.

In summary, although FMEA has been successfully applied across several domains of aviation engineering, there remains a critical need for more comprehensive models that:

- Address multi-expert hesitation,
- Handle high-dimensional uncertainty,
- Reflect behavioural aspects of human judgment, and
- Focus specifically on high-risk systems like aircraft landing subsystems.

In this context, it becomes necessary to integrate behavioural decision theories that can model human cognitive biases and subjective evaluations under risk. Prospect Theory offers a well-established behavioural framework that captures how decision makers perceive gains and losses relative to a reference point, and how they weigh probabilities in a non-linear way. These aspects are especially important in aviation risk assessment, where rare but catastrophic events tend to be overemphasized by experts, and evaluations are strongly influenced by perceived thresholds of acceptable safety. Therefore, incorporating Prospect Theory into the fuzzy decision-making framework allows for more realistic modelling of expert psychology, especially when combined with uncertainty-handling tools like q-ROHFS and ranking-based MCDM methods like MABAC. This forms the basis for the novel hybrid model proposed in this study.

### Motivation and proposed approach

Given the limitations of classical FMEA and the increasing complexity of aviation systems, there is a growing need for more robust, flexible, and cognitively aware risk assessment frameworks. In high-stakes domains such as aircraft landing systems, expert evaluations are often uncertain, hesitant, and influenced by subjective behavioural tendencies. The traditional models, which assume numerical certainty and rational consistency, fall short in capturing these essential characteristics of real-world decision-making.

Recent advances in decision theory and fuzzy set extensions offer promising tools to bridge this gap. On one hand, the q-rung orthopair hesitant fuzzy set (q-ROHFS) provides a powerful mathematical structure to represent expert hesitation, multiple possible judgments, and nonlinear uncertainty. On the other hand, Prospect Theory, a behavioural decision model, introduces the capability to model reference-dependent evaluations, loss aversion, and risk sensitivity—factors frequently observed in expert-based risk prioritization tasks. In aviation, where risk perception often varies depending on the phase of flight and the perceived severity of potential outcomes, incorporating such behavioural elements becomes especially valuable.

Motivated by these considerations, this study proposes a hybrid FMEA framework that integrates q-ROHFS, Prospect Theory, and the Multi-Attributive Border Approximation Area Comparison (MABAC) method. The novelty and strength of the proposed approach can be summarized as follows:

- q-ROHFS is utilized to represent expert assessments, enabling richer modelling of ambiguity, hesitancy, and inconsistency.
- Prospect theory is embedded into the MABAC structure to incorporate behavioural realism, thus enhancing the psychological accuracy of risk prioritization.
- An extended TOPSIS-based similarity measure is adopted to derive objective expert weights, improving aggregation fairness.
- The Best-Worst Method (BWM) is applied to determine criteria weights with fewer pairwise comparisons and higher consistency.
- A generalized q-ROHF Minkowski distance measure is introduced to provide a flexible and unified metric framework, encompassing Hamming and Euclidean distances as special cases, and allowing problem-specific tuning via the δ parameter. It enables the decision-makers to move beyond fixed metrics and adapt the model according to the characteristics of the hesitant fuzzy environment and the problem context.
- Finally, a real-world case study is conducted on aircraft landing systems to validate the applicability and performance of the proposed model.

Through this integrated structure, the proposed method addresses both technical uncertainty and human behavioural bias, offering a more realistic and comprehensive solution for failure mode risk prioritization in aviation safety.

## Preliminaries

### q-Rung orthopair hesitant fuzzy sets

#### Definition 1

^5^ Let $\:X$ be a fixed set. A hesitant fuzzy set $\:H$ on $\:X$ is defined as

1 $$\:H=\left\{(x,h\left(x\right)):x\in\:X\right\}$$

where $\:h\left(x\right)$ is a set of different values in $\:\left[\text{0,1}\right]$, representing the possible membership degrees of $\:x\in\:X$ to the set $\:H$.

#### Definition 2

^6^ Let $\:X$ be a fixed set, then a q-rung orthopair fuzzy set (q-ROFS) $\:A$ on $\:X$ is defined as

2 $$\:A=\left\{{({x}_{i},\left({\mu\:}_{A}\left({x}_{i}\right),{\nu\:}_{A}\left({x}_{i}\right),\right))}_{q}:{x}_{i}\in\:\right\},\:\:q\ge\:1$$

where $\:{\mu\:}_{A}\left({x}_{i}\right)$ and $\:{\nu\:}_{A}\left({x}_{i}\right)$ denote the q-rung membership and q-rung non-membership degrees, respectively and $\:0\le\:{\mu\:}_{A}\left({x}_{i}\right),{\nu\:}_{A}\left({x}_{i}\right)\le\:1\:$with the condition $\:{\left({\mu\:}_{A}\left({x}_{i}\right)\right)}^{q}+{\left({\nu\:}_{A}\left({x}_{i}\right)\right)}^{q}\le\:1$, $\:q\ge\:1$.

#### Definition 3

^7^ Let $\:X$ be a fixed set, then q-rung orthopair hesitant fuzzy set (q-ROHFS) $\:Q$ on $\:X$ is defined as

3 $$\:Q=\left\{{({x}_{i},\left(h\left({x}_{i}\right),g\left({x}_{i}\right)\right):{x}_{i}\in\:X)}_{q}\right\},\:\:q\ge\:1$$

where $\:h\left({x}_{i}\right)$ and $\:g\left({x}_{i}\right)$ are sets of different values in $\:\left[\text{0,1}\right]$, referred to as the q-rung orthopair hesitant fuzzy membership and non-membership degrees, respectively. These satisfy the following conditions: for $\:\mu\:\in\:h\left({x}_{i}\right)$, $\:\nu\:\in\:g\left({x}_{i}\right);\:0\le\:\mu\:,\nu\:\le\:1\:and\:\:{\left({\mu\:}^{+}\right)}^{q}+{\left({\nu\:}^{+}\right)}^{q}\le\:1\:$where $\:\:\:{\mu\:}^{+}=\underset{\mu\:\in\:h\left({x}_{i}\right)}{{max}}\left\{\mu\:\right\},\:\:\:{\nu\:}^{+}=\underset{\nu\:\in\:h\left({x}_{i}\right)}{{max}}\left\{\nu\:\right\}$.

For convenience, q-rung orthopair hesitant fuzzy number (q-ROHFN) is denoted as $\:{(h\left(x\right),g\left(x\right))}_{q}$ where $\:h\left( x \right) = \left\{ {\mu \:_{1} ,\mu \:_{2} , \ldots \:,\mu \:_{{\# h\left( x \right)}} } \right\}$, $\:g\left(x\right)=\left\{{\nu\:}_{1},{\nu\:}_{2},\dots\:,{\nu\:}_{\#g\left(x\right)}\right\}$, and $\:\#h\left(x\right)$, $\:\#g\left(x\right)$ are the total numbers of elements in $\:h\left(x\right)$ and $\:g\left(x\right)$, respectively.

If $\:\#h\left(x\right)=\#g\left(x\right)=1$, then q-ROHFS reduces to q-ROFS.

#### Definition 4

^7^ Let $\:\alpha\:={({h}_{\alpha\:}\left(x\right),{g}_{\alpha\:}\left(x\right))}_{q}$, $\:{\alpha\:}_{1}={({h}_{{\alpha\:}_{1}}\left(x\right),{g}_{{\alpha\:}_{1}}\left(x\right))}_{q}$ and $\:{\alpha\:}_{2}={({h}_{{\alpha\:}_{2}}\left(x\right),{g}_{{\alpha\:}_{2}}\left(x\right))}_{q}$ be three q-ROHFNs and $\:\lambda\:>0$. Then the basic algebraic properties on q-ROHFNs are defined as follows:

i. $$\:\alpha \:_{1} \oplus \:\alpha \:_{2} = (\bigcup\limits_{\begin{subarray}{l} \mu \:_{{\alpha {\kern 1pt} _{1} }} \in \:h_{{\alpha {\kern 1pt} _{1} }} \left( x \right),\nu \:_{{\alpha {\kern 1pt} _{1} }} \in \:g_{{\alpha {\kern 1pt} _{1} }} \left( x \right) \\ \mu \:_{{\alpha {\kern 1pt} _{2} }} \in \:h_{{\alpha {\kern 1pt} _{2} }} \left( x \right),\nu \:_{{\alpha {\kern 1pt} _{2} }} \in \:g_{{\alpha {\kern 1pt} _{2} }} \left( x \right) \end{subarray} } {\left\{ {\left( {\mu \:_{{\alpha \:_{1} }}^{q} + \mu \:_{{\alpha \:_{2} }}^{q} - \mu \:_{{\alpha \:_{1} }}^{q} \mu \:_{{\alpha \:_{2} }}^{q} } \right)^{{\frac{1}{q}}} ,\left( {\nu \:_{{\alpha \:_{1} }} \nu \:_{{\alpha \:_{2} }} } \right)} \right\}} )_{q}$$
ii. $$\:{\alpha\:}_{1} \otimes {\alpha\:}_{2}={(\bigcup\:_{\genfrac{}{}{0pt}{}{{\mu\:}_{{\alpha\:}_{1}}\in\:{h}_{{\alpha\:}_{1}}\left(x\right),{\nu\:}_{{\alpha\:}_{1}}\in\:{g}_{{\alpha\:}_{1}}\left(x\right)}{{\mu\:}_{{\alpha\:}_{2}}\in\:{h}_{{\alpha\:}_{2}}\left(x\right),{\nu\:}_{{\alpha\:}_{2}}\in\:{g}_{{\alpha\:}_{2}}\left(x\right)}}\left\{\left({\mu\:}_{{\alpha\:}_{1}}{\mu\:}_{{\alpha\:}_{2}}\right),{\left({\nu\:}_{{\alpha\:}_{1}}^{q}+{\nu\:}_{{\alpha\:}_{2}}^{q}-{\nu\:}_{{\alpha\:}_{1}}^{q}{\nu\:}_{{\alpha\:}_{2}}^{q}\right)}^{\frac{1}{q}}\right\})}_{q}$$(4)
iii. $$\:\lambda\:\alpha\:=\bigcup\:_{{\mu\:}_{\alpha\:}\in\:{h}_{\alpha\:}\left(x\right),{\nu\:}_{\alpha\:}\in\:{g}_{\alpha\:}\left(x\right)}{(\left\{{\left(1-{\left(1-{\mu\:}_{\alpha\:}^{q}\right)}^{\lambda\:}\right)}^{\frac{1}{q}}\right\},\left\{{\nu\:}_{\alpha\:}^{\lambda\:}\right\})}_{q}$$
iv. $$\:{\alpha\:}^{\lambda\:}=\bigcup\:_{{\mu\:}_{\alpha\:}\in\:{h}_{\alpha\:}\left(x\right),{\nu\:}_{\alpha\:}\in\:{g}_{\alpha\:}\left(x\right)}{(\left\{{\mu\:}_{\alpha\:}^{\lambda\:}\right\},\left\{{\left(1-{\left(1-{\nu\:}_{\alpha\:}^{q}\right)}^{\lambda\:}\right)}^{\frac{1}{q}}\right\})}_{q}$$

#### **Definition 5**

^7^ Let $\:\alpha\:={({h}_{\alpha\:}\left(x\right),{g}_{\alpha\:}\left(x\right))}_{q}$ be a q-ROHFN where $\:{h}_{\alpha\:}\left(x\right)=\left\{\bigcup\:_{r=1}^{\#{h}_{\alpha\:}\left(x\right)}{\mu\:}_{\alpha\:}^{r}\right\}$ and $\:{g}_{\alpha\:}\left(x\right)=\left\{\bigcup\:_{r=1}^{\#{g}_{\alpha\:}\left(x\right)}{\nu\:}_{\alpha\:}^{r}\right\}$. Then the score and accuracy function on q-ROHFN is defined as

5 $$\:S\left(\alpha\:\right)=\frac{1}{\#{h}_{\alpha\:}\left(x\right)}\sum\:_{r=1}^{\#{h}_{\alpha\:}\left(x\right)}{\mu\:}_{\alpha\:}^{r}-\frac{1}{\#{g}_{\alpha\:}\left(x\right)}\sum\:_{r=1}^{\#{g}_{\alpha\:}\left(x\right)}{\nu\:}_{\alpha\:}^{r}$$

and

6 $$\:A\left(\alpha\:\right)=\frac{1}{\#{h}_{\alpha\:}\left(x\right)}\sum\:_{r=1}^{\#{h}_{\alpha\:}\left(x\right)}{\mu\:}_{\alpha\:}^{r}+\frac{1}{\#{g}_{\alpha\:}\left(x\right)}\sum\:_{r=1}^{\#{g}_{\alpha\:}\left(x\right)}{\nu\:}_{\alpha\:}^{r}$$

respectively.

#### Definition 6

^7^ Let $\:{\alpha\:}_{1}={({h}_{{\alpha\:}_{1}}\left(x\right),{g}_{{\alpha\:}_{1}}\left(x\right))}_{q}$ and $\:{\alpha\:}_{2}={({h}_{{\alpha\:}_{2}}\left(x\right),{g}_{{\alpha\:}_{2}}\left(x\right))}_{q}$ be two q-ROHFNs. Then.

i. If $$\:S\left({\alpha\:}_{1}\right)<S\left({\alpha\:}_{2}\right)$$, then $$\:{\alpha\:}_{1}\prec\:{\alpha\:}_{2}$$.
ii. If $\:S\left({\alpha\:}_{1}\right)=S\left({\alpha\:}_{2}\right)$, then.

- If $$\:A\left({\alpha\:}_{1}\right)<A\left({\alpha\:}_{2}\right)$$, then $$\:{\alpha\:}_{1}\prec\:{\alpha\:}_{2}$$.
- If $$\:A\left({\alpha\:}_{1}\right)=A\left({\alpha\:}_{2}\right)$$, then $$\:{\alpha\:}_{1}\approx\:{\alpha\:}_{2}$$.

#### Definition 7

^45^ Let $\:X\ne\:{\varnothing}$ and $\:{\alpha\:}_{j}={\left({h}_{{\alpha\:}_{j}}\left(x\right),{g}_{{\alpha\:}_{j}}\left(x\right)\right)}_{q}$ be a collection of q-ROHFNs on $\:X$. Then q-rung orthopair hesitant fuzzy weighted averaging (q-ROHFWA) operator is defined as

7 $$\begin{aligned} & q - ROHFWA\left( {\alpha \:_{1} ,\alpha \:_{2} , \ldots \:,\alpha \:_{n} } \right) = w_{1} \alpha \:_{1} \oplus \:w_{2} \alpha \:_{2} \oplus \: \cdots \: \oplus \:w_{n} \alpha \:_{n} \\ & \quad = \bigcup {\:_{{\mu \:_{j} \in \:h_{{\alpha {\kern 1pt} _{j} }} ,\nu \:_{j} \in \:g_{{\alpha {\kern 1pt} _{j} }} }} } \left\{ {\left\{ {\left( {1 - \prod {\:_{{j = 1}}^{n} } \left( {1 - \mu \:_{j}^{q} } \right)^{{w_{j} }} } \right)^{{1/q}} } \right\},\left\{ {\prod {\:_{{j = 1}}^{n} } \nu \:_{j}^{{w_{j} }} } \right\}} \right\} \\ \end{aligned}$$

$$\:\text{w}\text{h}\text{e}\text{r}\text{e}\:{w}_{j}\in\:\left[\text{0,1}\right]\:\text{a}\text{n}\text{d}\:\sum\:_{j=1}^{n}{w}_{j}=1.$$.

#### Definition 8

^45^ Let $\:X\ne\:{\varnothing}$ and $\:{\alpha\:}_{j}={\left({h}_{{\alpha\:}_{j}}\left(x\right),{g}_{{\alpha\:}_{j}}\left(x\right)\right)}_{q}$ be a collection of q-ROHFNs on $\:X$. Then q-rung orthopair hesitant fuzzy weighted geometric (q-ROHFWG) operator is defined as

8 $$\begin{aligned} & q - ROHFWG\left( {\alpha \:_{1} ,\alpha \:_{2} , \ldots \:,\alpha \:_{n} } \right) = \alpha \:_{1}^{{w_{1} }} \otimes \:\alpha \:_{2}^{{w_{2} }} \otimes \: \cdots \: \otimes \:\alpha \:_{n}^{{w_{n} }} \\ & \quad = \bigcup {\:_{{\mu \:_{j} \in \:h_{{\alpha {\kern 1pt} _{j} }} ,\nu \:_{j} \in \:g_{{\alpha {\kern 1pt} _{j} }} }} } \left\{ {\left\{ {\prod {\:_{{j = 1}}^{n} } \mu \:_{j}^{{w_{j} }} } \right\},\left\{ {\left( {1 - \prod {\:_{{j = 1}}^{n} } \left( {1 - \nu \:_{j}^{q} } \right)^{{w_{j} }} } \right)^{{1/q}} } \right\}} \right\} \\ \end{aligned}$$

$$\:\text{w}\text{h}\text{e}\text{r}\text{e}\:{w}_{j}\in\:\left[\text{0,1}\right]\:\text{a}\text{n}\text{d}\:\sum\:_{j=1}^{n}{w}_{j}=1.$$.

#### Definition 9

Let $\:Q$ be the set of Q-ROHFSs. Then $\:d:Q\times\:Q\to\:\mathcal{R}$ is called Q-rung hesitant fuzzy distance measure if the following conditions are satisfied for $\:A,\:B,\:C\in\:Q$,

i. $$\:d\left(A,B\right)\ge\:0$$
ii. $\:d\left(A,B\right)=0$ if and only if $\:A=B$;
iii. $$\:d\left(A,B\right)=d\left(B,A\right)$$
iv. If $\:A\subseteq\:B\subseteq\:C$, then $\:d\left(A,C\right)\ge\:d\left(A,B\right)$ and $\:d\left(A,C\right)\ge\:d\left(B,C\right)$.

A novel distance measure is defined to be used in this paper as the following definition:

#### Definition 10

Let $\:X=\left\{{x}_{1},{x}_{2},\dots\:,{x}_{n}\right\}$ be a fixed set and $\:A=\left\{{({x}_{i},\left({h}_{A}\left({x}_{i}\right),{g}_{A}\left({x}_{i}\right)\right):{x}_{i}\in\:X)}_{q}\right\}$ and $\:B=\left\{{({x}_{i},\left({h}_{B}\left({x}_{i}\right),{g}_{B}\left({x}_{i}\right)\right):{x}_{i}\in\:X)}_{q}\right\}$ with $\:q\ge\:1$ be two Q-ROHFSs where $\:{h}_{A}\left(x\right)=\left\{\bigcup\:_{r=1}^{\#{h}_{A}\left(x\right)}{\mu\:}_{A}^{r}\right\}$, $\:{g}_{A}\left(x\right)=\left\{\bigcup\:_{r=1}^{\#{g}_{A}\left(x\right)}{\nu\:}_{A}^{r}\right\}$ and $\:{h}_{B}\left(x\right)=\left\{\bigcup\:_{r=1}^{\#{h}_{B}\left(x\right)}{\mu\:}_{B}^{r}\right\}$, $\:{g}_{B}\left(x\right)=\left\{\bigcup\:_{r=1}^{\#{g}_{B}\left(x\right)}{\nu\:}_{B}^{r}\right\}$. Then

9 $$\begin{gathered} \:d_{Q} \left( {A,B} \right) = \frac{1}{n}\left[ {\sum {\:_{{i = 1}}^{n} } \frac{1}{4}} \right.\left( {\left( {\frac{1}{{\# h_{A} \left( {x_{i} } \right)}}\sum {\:_{{\mu \:_{A}^{r} \in \:h_{A} \left( {x_{i} } \right)}} } \mathop {min}\limits_{{\mu \:_{B}^{r} \in \:h_{B} \left( {x_{i} } \right)}} \left| {\mu \:_{A}^{r} - \mu \:_{B}^{r} } \right|^{{\delta \:}} } \right.} \right. \hfill \\ \left. { + \frac{1}{{\# g_{A} \left( {x_{i} } \right)}}\sum {\:_{{\nu \:_{A}^{r} \in \:g_{A} \left( {x_{i} } \right)}} } \mathop {min}\limits_{{\nu \:_{B}^{r} \in \:g_{B} \left( {x_{i} } \right)}} \left| {\nu \:_{A}^{r} - \nu \:_{B}^{r} } \right|^{{\delta \:}} } \right)^{{1/\delta \:}} \hfill \\ + \left( {\frac{1}{{\# h_{B} \left( x \right)}}\sum {\:_{{\mu \:_{B}^{r} \in \:h_{B} \left( {x_{i} } \right)}} } \mathop {min}\limits_{{\mu \:_{A}^{r} \in \:h_{A} \left( {x_{i} } \right)}} \left| {\mu \:_{B}^{r} - \mu \:_{A}^{r} } \right|^{{\delta \:}} } \right. \hfill \\ \left. {\left. {\left. { + \frac{1}{{\# g_{B} \left( {x_{i} } \right)}}\sum {\:_{{\nu \:_{B}^{r} \in \:g_{B} \left( {x_{i} } \right)}} } \mathop {min}\limits_{{\nu \:_{A}^{r} \in \:h_{A} \left( {x_{i} } \right)}} \left| {\nu \:_{B}^{r} - \nu \:_{A}^{r} } \right|^{{\delta \:}} } \right)^{{1/\delta \:}} } \right)} \right] \hfill \\ \end{gathered}$$

is called the generalized distance measure between $\:A$ and $\:B$.

It can be easily shown that $\:{d}_{Q}$ satisfies the distance measure conditions. If $\:\delta\:=1$ and $\:\delta\:=2$, we get the q-rung orthopair hesitant fuzzy Hamming distance $\:{d}_{H}$ and q-rung orthopair hesitant fuzzy Euclidian distance $\:{d}_{E}$, respectively.

### Classical FMEA model

Risk management plays a vital role across numerous areas, hence, conducting this process in a systematic and expert-guided manner is essential. Failure Mode and Effects Analysis (FMEA) is a widely recognized method that aims to identify potential failures within a system, thereby preventing various forms of damage ranging from economic losses to threats to human life. This method has been extensively applied in high-risk domains to address uncertainties related to system reliability. When potential damages are identified and preventive actions are implemented in advance, this not only minimizes losses but also significantly enhances the reliability of the respective company, institution, or organization. The classical FMEA approach follows a structured sequence of steps, as outlined below:

Step 1: Define the entire process.

Step 2: Form a multidisciplinary FMEA team.

Step 3: Gather data on potential failure modes that may occur during the process, and classify related risks under three main categories: Severity (S), Occurrence (O), and Detection (D).

- Severity reflects the seriousness of the consequences of a failure mode.
- Occurrence indicates the frequency or likelihood of the failure mode happening.
- Detection refers to the ability to detect the failure before it reaches the end user.

Step 4: Evaluate each failure mode according to these risk factors.

Step 5: Calculate the Risk Priority Number (RPN) as the product of the three evaluations

10 $$\:RPN=O\times\:S\times\:D$$

The higher the RPN value, the higher the priority of that particular failure mode, necessitating more immediate corrective actions. In the classical FMEA model, the values for severity, occurrence, and detection are assigned by experts using a scale from $\:1$ to $\:10$, which results in an RPN value ranging from $\:1$ to $\:1000$. The mitigation strategies are then planned and implemented based on the magnitude of the calculated RPN values.

### Prospect theory

Prospect theory, originally proposed by Kahneman and Tversky^15^, is based on the idea that human psychology significantly influences decision-making under conditions of risk, particularly in contexts involving potential gains and losses. Unlike classical utility theory, which assumes rational and linear responses, prospect theory acknowledges that individuals evaluate outcomes relative to a reference point and that the value function behaves differently for gains and losses.

In particular, human responses to gains are generally concave, indicating risk aversion, while responses to losses are typically convex, reflecting risk-seeking behaviour. Moreover, losses are perceived to be more impactful than gains of the same magnitude—a phenomenon known as loss aversion. The overall prospect value $\:V$ is calculated using a value function $\:\nu\:\left({\varDelta\:x}_{i}\right)$ and a probability weighting function $\:w\left({p}_{i}\right)$ as follows:

11 $$\:V=\sum\:_{i=1}^{n}w\left({p}_{i}\right)\nu\:\left({\varDelta\:x}_{i}\right),\:\:\left({\varDelta\:x}_{i}={x}_{i}-{x}_{0}\right)$$

Here $\:{\:x}_{0}$denotes the reference point, and $\:{\varDelta\:x}_{i}$ is the deviation of the outcome $\:{x}_{i}$ from that reference. The value and probability weight functions are defined as

12 $$\:\left({\varDelta\:x}_{i}\right)=\left\{\begin{array}{cc}{\left(\varDelta\:x\right)}^{\alpha\:},&\:\varDelta\:x\ge\:0\\\:-\theta\:{\left(-\varDelta\:x\right)}^{\beta\:}&\:\varDelta\:x<0\end{array}\right.\:and\:w\left({p}_{i}\right)=\left\{\begin{array}{cc}\frac{{p}^{\gamma\:}}{{\left({p}^{\gamma\:}+{\left(1-p\right)}^{\gamma\:}\right)}^{\raisebox{1ex}{$1$}\!\left/\:\!\raisebox{-1ex}{$\gamma\:$}\right.}}&\:\varDelta\:x\ge\:0\\\:\frac{{p}^{\delta\:}}{{\left({p}^{\delta\:}+{\left(1-p\right)}^{\delta\:}\right)}^{\raisebox{1ex}{$1$}\!\left/\:\!\raisebox{-1ex}{$\delta\:$}\right.}}&\:\varDelta\:x<0\end{array}\right.$$

where $\:\alpha\:$ and $\:\beta\:$ are sensitivity coefficients for gains and losses, $\:\theta\:$ represents the loss aversion coefficient, and $\:\gamma\:$ and $\:\delta\:$ are risk gain and risk loss coefficients, respectively. For optimal solutions, the commonly used parameter values for optimal representation are

$$\:\alpha\:=\beta\:=0.88,\:\theta\:=2.25\:\text{a}\text{n}\text{d}\:\gamma\:=0.61,\:\delta\:=0.72.$$

These functions enable the integration of human cognitive biases into decision-making models, making prospect theory especially suitable for domains where uncertainty, subjective judgment, and emotional weighting of outcomes are significant such as failure risk assessment in complex engineering systems.

## Proposed FMEA model

Failure Mode and Effects Analysis is an effective tool for identifying known or potential deficiencies and prioritizing risks within a system. Even minor failures can result in significant damage; therefore, appropriate preventive measures must be taken before systems become operational. FMEA has found widespread application in military, industrial, and medical domains, among others. However, to overcome some of the limitations of the classical FMEA approach, the problem is reformulated as a multi-criteria decision-making (MCDM) task. In this study, a novel FMEA model is proposed by integrating the MABAC method under a q-Rung Orthopair Hesitant Fuzzy Set (Q-ROHFS) environment. The weights of the risk factors, namely, Occurrence (O), Severity (S), and Detection (D) are determined using the Best-Worst Method (BWM). First, all failure modes $\:{FM}_{i}$ are identified and evaluated according to the risk factors $\:{R}_{j}$, categorized under the O, S, and D dimensions. To improve the prioritization process, the MABAC method is employed to assess the relative importance of each failure mode within the FMEA framework.

Let $\:{N}^{k}=\left({N}_{ij}^{k}\right)$ represent the decision matrix related to the assessments of failure modes according to the risk factors under the categories (O), (S) and (D) given by each expert in accordance with Q-ROHFNs. Here $\:{N}_{ij}^{k}$ is evaluation of i$\:th$ failure mode $\:{FM}_{i}$ according to the j$\:th$ risk factor $\:{R}_{j}$ given by k$\:th$ decision maker $\:{D}_{k}$ and $\:{N}_{ij}^{k}$ is the form of Q-ROHFNs as $\:{N}_{ij}^{k}=\left({\left({h}_{{N}_{ij}^{k}},{g}_{{N}_{ij}^{k}}\right)}_{q}\right)$ with $\:{h}_{{N}_{ij}^{k}}=\left\{\bigcup\:_{r=1}^{\#{h}_{{N}_{ij}^{k}}}{\mu\:}_{{N}_{ij}^{k}}^{r}\right\}$, $\:{g}_{{N}_{ij}^{k}}=\left\{\bigcup\:_{r=1}^{\#{g}_{{N}_{ij}^{k}}}{\nu\:}_{{N}_{ij}^{k}}^{r}\right\}$ for $\:i=\text{1,2},\dots\:,n;\:j=\text{1,2},\dots\:,m;k=\text{1,2},\dots\:,l$.

### Determination of decision maker’s weights

In any group decision-making process, it is essential to reflect the expertise and reliability of decision makers in an objective and systematic way to ensure that the final outcome is both effective and efficient. To achieve this, the present study utilizes the principles of the TOPSIS method introduced by Hwang and Yoon^10^, in order to compute the relative weights of each decision maker. The following steps outline the process for determining the weights of decision makers:

Step 1: Construct the positive ideal decision matrix (PIDM) $\:{N}^{+}$ and negative ideal decision matrix (NIDM) $\:{N}^{-}$ defined as

13 $$\begin{gathered} \:N^{ + } \: = \left( {N_{{ij}}^{ + } } \right) = \left( {\left( {h_{{N_{{ij}}^{ + } }} ,g_{{N_{{ij}}^{ + } }} } \right)_{q} } \right) \hfill \\ \:N^{ - } = \left( {N_{{ij}}^{ - } } \right) = \left( {\left( {h_{{N_{{ij}}^{ - } }} ,g_{{N_{{ij}}^{ - } }} } \right)_{q} } \right) \hfill \\ \end{gathered}$$

where

14 $$\begin{gathered} \:h_{{N_{{ij}}^{ + } }} = \left\{ {\bigcup {\:_{{k = {\text{1,2}}, \ldots \:,l}} } \left\{ {\mathop {max}\limits_{{r = {\text{1,2}}, \ldots \:,\# h_{{N_{{ij}}^{k} }} }} \left( {\mu \:_{{N_{{ij}}^{k} }}^{r} } \right)} \right\}} \right\},\:g_{{N_{{ij}}^{ + } }} \hfill \\ = \left\{ {\bigcup {\:_{{k = {\text{1,2}}, \ldots \:,l}} } \left\{ {\mathop {min}\limits_{{r = {\text{1,2}}, \ldots \:,\# g_{{N_{{ij}}^{k} }} }} \left( {\nu \:_{{N_{{ij}}^{k} }}^{r} } \right)} \right\}} \right\}\:h_{{N_{{ij}}^{ - } }} \hfill \\ = \left\{ {\bigcup {\:_{{k = {\text{1,2}}, \ldots \:,l}} } \left\{ {\mathop {min}\limits_{{r = {\text{1,2}}, \ldots \:,\# h_{{N_{{ij}}^{k} }} }} \left( {\mu \:_{{N_{{ij}}^{k} }}^{r} } \right)} \right\}} \right\},\:g_{{N_{{ij}}^{ - } }} \hfill \\ = \left\{ {\bigcup {\:_{{k = {\text{1,2}}, \ldots \:,l}} } \left\{ {\mathop {max}\limits_{{r = {\text{1,2}}, \ldots \:,\# g_{{N_{{ij}}^{k} }} }} \left( {\nu \:_{{N_{{ij}}^{k} }}^{r} } \right)} \right\}} \right\} \hfill \\ \end{gathered}$$

for $$\:i=\text{1,2},\dots\:,n;\:j=\text{1,2},\dots\:,m$$.

Step 2: Compute the distance between each decision matrix $\:{N}^{k}$ given by k$\:th$ decision maker $\:{D}_{k}$ $\:\left(k=\text{1,2},\dots\:,l\right)$ and PIDM, NIDM, respectively as

15 $$\begin{gathered} \:d_{k}^{ + } = d_{H} \left( {N^{k} ,N^{ + } } \right) \hfill \\ \:d_{k}^{ - } = d_{H} \left( {N^{k} ,N^{ - } } \right) \hfill \\ \end{gathered}$$

where $\:{d}_{H}$ denotes the Q-ROHFS-based Hamming distance using (9).

Step 3: Obtain the positive and negative similarity degrees for each decision maker.

16 $$\begin{gathered} \:S_{k}^{ + } = 1 - \frac{{d_{k}^{ + } }}{{\sum {\:_{{k = 1}}^{l} } d_{k}^{ + } }} \hfill \\ \:S_{k}^{ - } = 1 - \frac{{d_{k}^{ - } }}{{\sum {\:_{{k = 1}}^{l} } d_{k}^{ - } }} \hfill \\ \end{gathered}$$

Step 4: Calculate the similarity degrees of each decision matrix with taking into consideration the decision maker’s psychological behaviour as

17 $$\:{S}_{k}=\alpha\:{S}_{k}^{+}+\left(1-\alpha\:\right){S}_{k}^{-}$$

If the decision maker is optimistic, $\:\alpha\:$ is close to $\:1$, otherwise, it is close to $\:0$.

Step 5: Finally, determine the weights of the each decision maker $\:{\lambda\:}_{k}$ as

18 $$\:{\lambda\:}_{k}=\frac{{S}_{k}}{{\sum\:}_{k=1}^{l}{S}_{k}},\:k=\text{1,2},\dots\:,l$$

### Determining weight of risk factors

In this paper, the weights of risk factors are determined using best-worst method (BMW) proposed by Rezaei^46^ under the Q-ROHF environment. This approach allows each decision maker’s subjective perception of the relative importance of criteria to be incorporated into the evaluation process, thereby enhancing flexibility and realism in decision-making. The procedure consists of the following steps:

Step 1: Identify the best and the worst criterion according to each decision maker. In this step, each decision maker expresses his/her best and worst option for criteria.

Step 2: Construct the preference evaluation vector for the best over others as $\:{C}_{B}^{k}=\left({C}_{B1}^{k},{C}_{B2}^{k},\dots\:,{C}_{Bm}^{k}\right)$. Here $\:{C}_{Bj}^{k}$ represents the preference values of the best criterion over the criterion $\:{C}_{j}$ according to the $\:k.$ decision maker. Each decision maker rates the best criterion over others with number between $\:1$ and $\:9$ (1= equally important: 9=extremely more important) and it is taken as $\:{C}_{BB}^{k}=1$.

Step 3: Determine the preference evaluation vector for all vectors over the worst as $\:{C}_{W}^{k}=\left({C}_{1W}^{k},{C}_{2W}^{k},\dots\:,{C}_{mW}^{k}\right)$. Here $\:{C}_{jW}^{k}$ represents the preference values of all criteria over the worst according to the $\:k.$ decision maker. Each decision maker rates all criteria over the worst with number between $\:1$ and $\:9$ and it is taken as $\:{C}_{WW}^{k}=1$.

Step 4: Determine optimal criteria weights $\:\left({w}_{1}^{k\text{*}},{w}_{2}^{k\text{*}},\dots\:,{w}_{m}^{k\text{*}}\right)$ relative to each decision maker $\:{D}^{k}$ $\:\left(k=\text{1,2},\dots\:,l\right)$ by taking each pairs $\:\frac{{w}_{B}^{k}}{{w}_{j}^{k}}$ and $\:\frac{{w}_{j}^{k}}{{w}_{W}^{k}}$ as $\:{C}_{Bj}^{k}=\frac{{w}_{B}^{k}}{{w}_{j}^{k}}$ and $\:{C}_{jW}^{k}=\frac{{w}_{j}^{k}}{{w}_{W}^{k}}$. To obtain optimal weights for each decision maker $\:\left(k=\text{1,2},\dots\:,l\right)$, we minimize the maximum absolute differences as

19 $$\:min\mathop {max\:}\limits_{j} \left\{ {\left\lfloor {\frac{{w_{B}^{k} }}{{w_{j}^{k} }} - C_{{Bj}}^{k} } \right\rfloor ,\left\lfloor {\frac{{w_{j}^{k} }}{{w_{W}^{k} }} - C_{{jW}}^{k} } \right\rfloor } \right\}$$

$$\:where\:{w}_{j}^{k}\ge\:0\:\text{a}\text{n}\text{d}\:\:\sum\:_{j=1}^{m}{w}_{j}^{k}=1\:for\:all\:j.$$.

The problem in Eq. (19) can be considered as the optimization model given below

20 $$\:\left\{\begin{array}{c}\left|\frac{{w}_{B}^{k}}{{w}_{j}^{k}}-{C}_{Bj}^{k}\right|\le\:{\xi\:}^{k}\\\:\left|\frac{{w}_{j}^{k}}{{w}_{W}^{k}}-{C}_{jW}^{k}\right|\le\:{\xi\:}^{k}\\\:{w}_{j}^{k}\ge\:0\:and\:\sum\:_{j=1}^{m}{w}_{j}^{k}=1,for\:all\:j\:\end{array}\right.$$

With the solution of this optimization model, we can find optimal weights of risk factors $\:\left({w}_{1}^{k\text{*}},{w}_{2}^{k\text{*}},\dots\:,{w}_{m}^{k\text{*}}\right)$ and $\:{\left({\xi\:}^{k}\right)}^{\text{*}}$ for each decision maker. $\:{\left({\xi\:}^{k}\right)}^{\text{*}}$ represents the consistency of results. If $\:{\left({\xi\:}^{k}\right)}^{\text{*}}$ is close to zero, it presents a higher consistency. Otherwise, it means less consistency.

### Novel MABAC based on prospect theory under q-Rung orthopair hesitant fuzzy environment

**Fig. 1:**
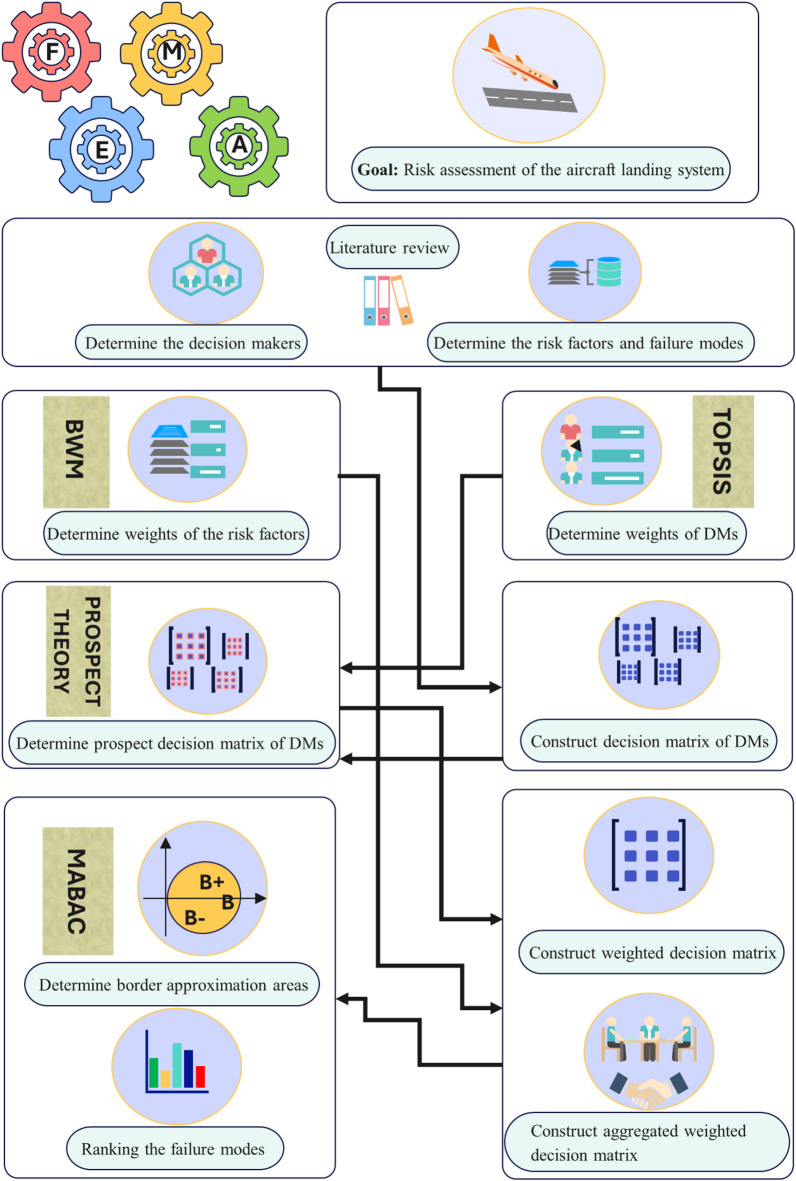
Flowchart of the proposed FMEA method.

Designing FMEA by considering multiple risk factors is of great importance to ensure more reliable and robust system performance. In this study, an extended FMEA approach is proposed by integrating the MABAC method with prospect theory under a q-Rung Orthopair Hesitant Fuzzy Set (Q-ROHFS) environment. The proposed method is applied to analyse and prioritize failure modes. After identifying all potential failure modes with the help of expert opinions and classify the evaluation criteria into three groups: Occurrence (O), Severity (S) and Detection (D), the complete framework of the novel MABAC method can be given as illustrated in Fig. 1, and its implementation steps are as follows:

Step 1: Construct decision matrix $\:{N}^{k}=\left({N}_{ij}^{k}\right)$ related to the assessments of failure modes according to the risk factors under the categories (O), (S) and (D) given by each expert in accordance with Q-ROHFNs.

Remind that $\:{N}_{ij}^{k}$ as stated above is evaluation of $\:{i}^{th}$ failure mode according to the $\:{j}^{th}$ risk factor given by $\:{k}^{th}$ decision maker and evaluation data $\:{N}_{ij}^{k}$ are q-rung Orthopair hesitant fuzzy number as $\:{N}_{ij}^{k}={\left({h}_{{N}_{ij}^{k}},{g}_{{N}_{ij}^{k}}\right)}_{q}$ with $\:{h}_{{N}_{ij}^{k}}=\bigcup\:_{r=1}^{\#{h}_{{N}_{ij}^{k}}}\left\{{\mu\:}_{{N}_{ij}^{k}}^{r}\right\}$, $\:{g}_{{N}_{ij}^{k}}=\bigcup\:_{r=}^{\#{g}_{{N}_{ij}^{k}}}\left\{{\nu\:}_{{N}_{ij}^{k}}^{r}\right\}$ for $\:i=\text{1,2},\dots\:,n;\:j=\text{1,2},\dots\:,m;k=\text{1,2},\dots\:,l$.

Step 2: Determine the weights of each expert $\:{\gamma\:}^{k}$ for $\:k=\text{1,2},\dots\:,l$ with the method given in “Determination of decision maker’s weights”.

**Step 3.** Construct the prospect decision matrix $\:{R}^{k}=\left({r}_{ij}^{k}\right)$ for each expert $\:\left(k=\text{1,2},\dots\:,l\right)$ by incorporating their psychological reference points and stability attitudes as follows:

21 $$\:{r}_{ij}^{k}=\pi\:\left({p}_{j}^{k}\right)V\left({N}_{ij}^{k}\right),\:i=\text{1,2},\dots\:,n;\:j=\text{1,2},\dots\:,m$$

where the value function $\:V\left({N}_{ij}^{k}\right)$ and the probability weighting function $\:\pi\:\left({p}_{j}^{k}\right)$ are defined as:

22 $$\:V\left({N}_{ij}^{k}\right)=\left\{\begin{array}{cc}{\left({d}_{H}\left({N}_{ij}^{k},{N}_{j}^{k}\right)\right)}^{\alpha\:},&\:{N}_{ij}^{k}\ge\:{N}_{j}^{k}\\\:-\theta\:{\left(-{d}_{H}\left({N}_{ij}^{k},{N}_{j}^{k}\right)\right)}^{\beta\:},&\:{N}_{ij}^{k}<{N}_{j}^{k}\end{array}\right.$$

and

23 $$\:\pi\:\left({p}_{j}^{k}\right)\:=\left\{\begin{array}{cc}\frac{{\left({p}_{j}^{k}\right)}^{\gamma\:}}{{\left({\left({p}_{j}^{k}\right)}^{\gamma\:}+{\left(1-{p}_{j}^{k}\right)}^{\gamma\:}\right)}^{\raisebox{1ex}{$1$}\!\left/\:\!\raisebox{-1ex}{$\gamma\:$}\right.}},&\:{N}_{ij}^{k}\ge\:{N}_{j}^{k}\\\:\frac{{\left({p}_{j}^{k}\right)}^{\delta\:}}{{\left({\left({p}_{j}^{k}\right)}^{\delta\:}+{\left(1-{p}_{j}^{k}\right)}^{\delta\:}\right)}^{\raisebox{1ex}{$1$}\!\left/\:\!\raisebox{-1ex}{$\delta\:$}\right.}},&\:{N}_{ij}^{k}<{N}_{j}^{k}\end{array}\right.$$

Here $\:\gamma\:$ and $\:\delta\:$ are coefficients of risk gain and risk loss, respectively. For optimal solution, they are taken as $\:\alpha\:=\beta\:=0.88,\:\theta\:=2.25$ and $\:\gamma\:=0.61$, $\:\delta\:=0.72$. Also, $\:{p}_{j}^{k}$ is determined by the probability density function which formed with normal distribution as

24 $$\:{p}_{j}^{k}=\frac{{e}^{-\frac{{\left(S\left({N}_{ij}^{k}\right)-S\left({\tau\:}_{j}^{k}\right)\right)}^{2}}{2{\left(S\left({\sigma\:}_{j}^{k}\right)\right)}^{2}}}}{\sum\:_{i=1}^{n}{e}^{-\frac{{\left(S\left({N}_{ij}^{k}\right)-S\left({\tau\:}_{j}^{k}\right)\right)}^{2}}{2{\left(S\left({\sigma\:}_{j}^{k}\right)\right)}^{2}}}}$$

where $\:{\tau\:}_{j}^{k}$ and $\:{\sigma\:}_{j}^{k}$ are mean value and standard deviation of $\:{\left\{{N}_{ij}^{k}\right\}}_{i}$ $\:\left(j=\text{1,2},\dots\:,m\right)$ for each decision maker, respectively:

25 $$\:{\tau\:}_{j}^{k}=\left(\frac{1}{n}\sum\:_{i=1}^{n}\left(\frac{1}{\#{h}_{{N}_{ij}^{k}}}\sum\:_{r=1}^{\#{h}_{{N}_{ij}^{k}}}{\mu\:}_{{N}_{ij}^{k}}^{r}\right),\frac{1}{n}\sum\:_{i=1}^{n}\left(\frac{1}{\#{g}_{{N}_{ij}^{k}}}\sum\:_{r=1}^{\#{g}_{{N}_{ij}^{k}}}{\nu\:}_{{N}_{ij}^{k}}^{r}\right)\right)$$

and

26 $$\:{\sigma\:}_{j}^{k}={\left(\frac{1}{n}\sum\:_{i=1}^{n}{\left(S\left({N}_{ij}^{k}\right)-S\left({\tau\:}_{j}^{k}\right)\right)}^{2}\right)}^{1/2}$$

Step 4: Calculate the weights of the risk factors $\:{w}_{j}^{k}$ for each expert using the BWM procedure outlined in “Determining weight of risk factors”.

Step 5: Obtain the weighted decision matrix

27 $$\:{V}^{k}=\left({w}_{j}^{k}{r}_{ij}^{k}\right)$$

where $\:{w}_{j}^{k}$ is the weight of the j$\:th$ risk factor according to the k$\:th$ decision maker.

Step 6: Compute the aggregated weighted decision matrix $\:V=\left({t}_{ij}\right)$ using the $\:q-ROHFWA$ or $\:q-ROHFWG$ operators as

28 $$\:V=q-ROHFWA\left({V}^{1},{V}^{2},\dots\:,{V}^{l}\right)=\bigcup\:_{{\mu\:}_{k}\in\:{h}_{{\alpha\:}_{k}},{\nu\:}_{k}\in\:{g}_{{\alpha\:}_{k}}}\left\{\left\{{\left(1-\prod\:_{k=1}^{l}{\left(1-{\mu\:}_{k}^{q}\right)}^{{\gamma\:}^{k}}\right)}^{1/q}\right\},\left\{\prod\:_{k=1}^{l}{\nu\:}_{k}^{{\gamma\:}^{k}}\right\}\right\}$$

Step 7: Construct the border approximation area matrix $\:B=\left({b}_{j}\right)$ as the reference point for each expert by

29 $$\:{b}_{j}={\left(\prod\:_{i=1}^{n}{t}_{ij}\right)}^{\raisebox{1ex}{$1$}\!\left/\:\!\raisebox{-1ex}{$n$}\right.}for\:j=\text{1,2},\dots\:,m$$

Here $\:{b}_{j}$ denotes the border approximation value for $\:{j}^{th}$ risk factor and serves as a neutral reference for evaluating deviations in performance among alternatives.

Step 8: Compute the distance matrix $\:D=\left({d}_{ij}\right)$ between border approximation area matrix $\:{b}_{j}$ and aggregated weighted decision matrix $\:\left({t}_{ij}\right)$ $\:i=\text{1,2},\dots\:,n$; $\:j=\text{1,2},\dots\:,m$ as below

30 $$\:{d}_{ij}=\left\{\begin{array}{cc}{d}_{H}\left({t}_{ij},{b}_{j}\right),&\:{t}_{ij}>{b}_{j}\\\:0,&\:{t}_{ij}={b}_{j}\\\:-{d}_{H}\left({t}_{ij},{b}_{j}\right),&\:{t}_{ij}<{b}_{j}\end{array}\right.$$

According to the above calculation $\:i.$ Failure mode can be located in one of the following three regions:

- $\:{B}^{+}:$ Upper Approximation Area—indicating favourable (better) performance.
- $\:B:$ Border Approximation Area—indicating neutral or average performance.
- $\:{B}^{-}:$ Lower Approximation Area—indicating poor or unfavourable performance.

Thus, the failure modes located in the upper approximation area $\:{B}^{+}$ are considered the most critical and prioritized accordingly.

Step 9: Rank the failure modes according to the $\:{S}_{i}$ values by the following Eq. 

31 $$\:{S}_{i}=\sum\:_{j=1}^{m}{d}_{ij},\:\:\:\:\:i=\text{1,2},\dots\:,n$$

Failure modes are then ranked based on their corresponding $\:{S}_{i}$​ values. A higher score indicates higher risk severity and thus, a higher priority in the risk mitigation process. These steps are given in detain in Algorithm 1.

**Figure:**
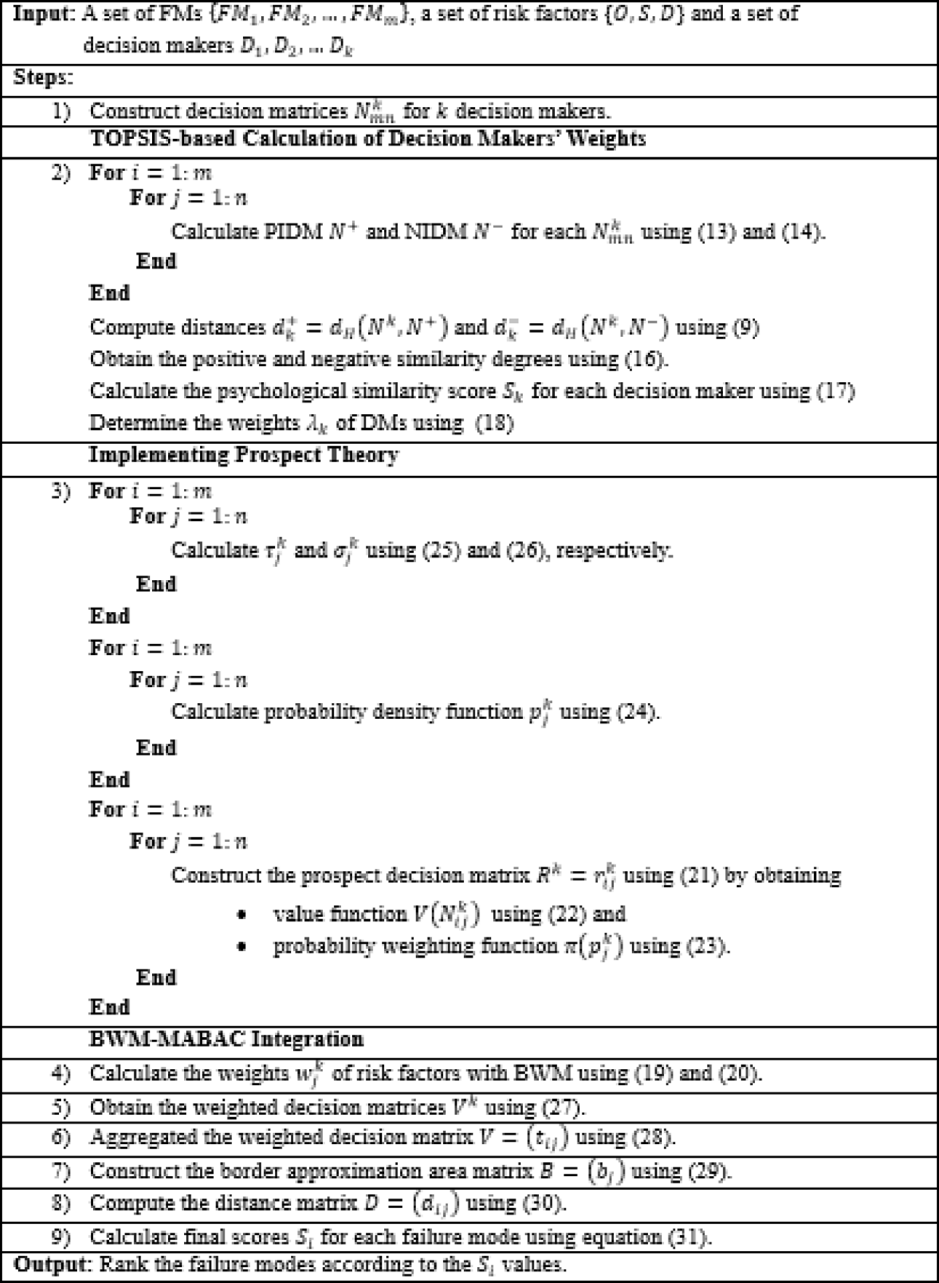
Algorithm 1. Proposed FMEA method.

## A case study at risk assessment problem of aircraft landing

The decision to utilize q-Rung Orthopair Hesitant Fuzzy Sets (q-ROHFSs) in the proposed FMEA model is driven by the complex and uncertain nature of real-world failure evaluations in aviation systems. For instance, failures such as “inconsistent extension of landing gear under variable weather conditions” or “intermittent brake response during high-speed taxi” often generate multiple, conflicting assessments among experts due to environmental variability and sensor ambiguity. Similarly, scenarios like “sensor malfunction due to undetected wiring degradation” or “hydraulic system pressure loss due to unobservable internal leaks” involve inherent uncertainty and hesitancy in expert judgments, as exact failure conditions may not be consistently replicable or observable. Moreover, human-centred issues such as “delayed reaction time in emergency gear override activation” further introduce subjective variability rooted in cognitive behaviour and procedural familiarity. These examples highlight that traditional crisp or even intuitionistic fuzzy models fall short in representing the depth of hesitation, duality of membership and non-membership, and expert inconsistency. Hence, q-ROHFSs provide a robust and flexible mathematical framework to capture such nuances, enabling more reliable and behaviour-sensitive risk prioritization in safety-critical systems.

For example, consider a potential failure mode such as “intermittent malfunction of the landing gear sensor during cabin temperature fluctuations.” An expert may assess the severity of this failure as high (e.g., $\:0.8$) yet may hesitate between several membership degrees such as $\:\{0.7,\:0.8,\:0.9\}$. At the same time, if this malfunction occurs very infrequently, the expert may also assign non-membership degrees such as $\:\{0.1,\:0.2\}$, taking into account the uncertainty in the data. Representing this evaluation in $\:q-ROHFS$ form for $\:q=2$, we may express it as:

$$\:A=\left(\left\{0.7,\:0.8,\:0.9\right\},\left\{0.1,\:0.2\right\}\right)$$

This structure enables the model to simultaneously handle multiple degrees of belief and disbelief with a adjustable parameter $\:q$, capturing both hesitation and decision-maker conservatism. For another failure mode such as “unexpected hydraulic pressure drops during landing,” evaluations by different experts may include overlapping and inconsistent values. For example,

$$\begin{gathered} \:Expert\:1 = \:\left( {\left\{ {{\text{0}}{\text{.6,0}}{\text{.7}}} \right\},\left\{ {0.3} \right\}} \right) \hfill \\ \:Expert\:2 = \:(\{ {\text{0}}{\text{.5,0}}{\text{.8}}\} ,\{ {\text{0}}{\text{.2,0}}{\text{.4}}\} ) \hfill \\ \end{gathered}$$

The q-ROHFS model aggregates these hesitant and orthopair evaluations meaningfully, avoiding the loss of nuanced opinions. These examples illustrate the necessity of employing q-ROHFNs over traditional crisp or even interval-valued fuzzy approaches, thereby ensuring a more flexible, psychologically realistic, and mathematically powerful modelling structure for FMEA in safety-critical systems.

### Numerical example

To test the applicability and practical effectiveness of the proposed q-ROHF-based MABAC method integrated with prospect theory, a real-world case study concerning the aircraft landing system is considered in this section. The case is originally detailed by Yazdi et al.^47^ who emphasized that malfunctions occurring during the landing phase can lead to critical operational failures and significant damage to the aircraft and its passengers. The landing process involves multiple complex subsystems, including gear extension, sensor control, fault reporting, and automated safety tests—all of which are highly sensitive to failure. Recognizing the high-risk nature of this process, they approached the problem as a structured risk management task and systematically identified ten potential failure modes $\:\left(F{M}_{i}\right),\:\left(i=1,\dots\:,10\right)$ that could occur during the landing sequence given in Table 1. Each of these failure modes has distinct effects on system safety and reliability. To assess the risk level associated with each failure mode, three domain experts were consulted to evaluate them in terms of three critical risk factors commonly used in FMEA: severity (S), occurrence (O) and detection (D). These assessments were expressed using q-Rung Orthopair Hesitant Fuzzy Numbers (q-ROHFNs) to capture the hesitation and uncertainty inherent in expert judgments, making the data suitable for advanced fuzzy decision analysis.

Importantly, the selection of experts followed the criteria suggested by Yazdi et al.^47^ where the importance of forming a heterogeneous expert group is emphasized to reflect varying experience levels, educational backgrounds, and professional roles. According to their approach, expert credibility was evaluated using measurable factors such as job experience, academic qualifications, age, and organizational position. This structure helps ensure that the opinions included in the assessment process are both diverse and reliable. The heterogeneous group setup adopted in this study enhances the realism of expert-based decision-making by incorporating different perspectives and reducing the risks associated with homogenous bias. This design also reflects the behavioural nature of real-world expert evaluations, where cognitive framing, subjectivity, and experiential differences often influence judgment.

**Table 1:** Possible failure modes of the landing systems.

Process step	Process step	Potential failure modes	Potential effect(s) of failure
FM1	Raising Gear	Fault in raising the wheels	Extension system damage of aircraft, taking off with open wheels
FM2		Raising wheels earlier than specified time	Aircraft damaged on landing
FM3	Coming down Gear	Fault in coming down the wheels	Landing with closed wheels, Aircraft damaged on landing
FM4		Coming down wheels earlier than specified time	Damage caused by the pressure and tension
FM5	Automatically test	Fault on the run	Possible unsafe conditions
FM6		Wrong run	Possible unsafe conditions
FM7	Fault reporting system	Fault on the run	Fault information was not reported, Without risk
FM8		Wrong run	Fault information was reported mistakenly, Without risk
FM9	Record the result of automatically test	Fault on the run	Failure to register unsafe situation, Without risk
FM10		Wrong run	Register unsafe situation mistakenly, Without risk

Step 1: The risk assessment values are given in Table 2 in the form of q-ROPHFNs.

**Table 2:** The q-ROHF decision matrices of decision makers.

Failure modes	DM1		
	O	S	D
FM1	$\:(\left(\text{0.9,0.1,0.4}\right),\left(\text{0.3,0.5}\right))$	$\:(\left(\text{0.4,0.4,0.2}\right),\left(\text{0.2,0.9}\right))$	$\:(\left(\text{0.8,0.7,0.5}\right),\left(\text{0.1,0.4}\right))$
FM2	$\:(\left(\text{0.3,0.5}\right),\left(\text{0.4,0.2,0.8}\right))$	$\:(\left(\text{0.8,0.3}\right),\left(\text{0.6,0.2,0.7}\right))$	$\:(\left(\text{0.6,0.5,0.2}\right),\left(\text{0.4,0.6,0.3}\right))$
FM3	$\:(\left(\text{0.7,0.3,0.6}\right),\left(\text{0.7,0.8,0.1}\right))$	$\:(\left(\text{0.1,0.2}\right),\left(\text{0.1,0.3}\right))$	$\:(\left(\text{0.7,0.1,0.7}\right),\left(\text{0.7,0.7}\right))$
FM4	$\:(\left(\text{0.4,0.1}\right),\left(\text{0.8,0.5}\right))$	$\:(\left(\text{0.1,0.4}\right),\left(\text{0.8,0.7,0.9}\right))$	$\:(\left(\text{0.9,0.4}\right),\left(\text{0.3,0.4}\right))$
FM5	$\:(\left(\text{0.8,0.2}\right),\left(\text{0.3,0.5}\right))$	$\:(\left(\text{0.1,0.2,0.4}\right),\left(\text{0.7,0.5,0.2}\right))$	$\:(\left(\text{0.6,0.9}\right),\left(\text{0.2,0.1}\right))$
FM6	$\:(\left(\text{0.6,0.4}\right),\left(\text{0.1,0.4}\right))$	$\:(\left(\text{0.2,0.7,0.5}\right),\left(\text{0.7,0.3,0.2}\right))$	$\:(\left(\text{0.5,0.8}\right),\left(\text{0.2,0.5,0.3}\right))$
FM7	$\:(\left(\text{0.5,0.9}\right),\left(\text{0.4,0.5,0.4}\right))$	$\:(\left(\text{0.6,0.8,0.7}\right),\left(\text{0.2,0.6,0.7}\right))$	$\:(\left(\text{0.1,0.8}\right),\left(\text{0.1,0.1,0.1}\right))$
FM8	$\:(\left(\text{0.4,0.4}\right),\left(\text{0.3,0.1}\right))$	$\:(\left(\text{0.8,0.2}\right),\left(\text{0.2,0.5}\right))$	$\:(\left(\text{0.9,0.1,0.8}\right),\left(\text{0.5,0.5,0.3}\right))$
FM9	$\:(\left(\text{0.1,0.8}\right),\left(\text{0.1,0.2,0.2}\right))$	$\:(\left(\text{0.1,0.4,0.4}\right),\left(\text{0.3,0.7,0.3}\right))$	$\:(\left(\text{0.5,0.2,0.4}\right),\left(\text{0.3,0.8,0.1}\right))$
FM10	$\:(\left(\text{0.2,0.4}\right),\left(\text{0.3,0.1,0.1}\right))$	$\:(\left(\text{0.2,0.7,0.7}\right),\left(\text{0.8,0.5,0.8}\right))$	$\:(\left(\text{0.1,0.6}\right),\left(\text{0.3,0.1}\right))$
	DM2		
FM1	$\:(\left(\text{0.8,0.7}\right),\left(\text{0.5,0.4}\right))$	$\:(\left(\text{0.7,0.3}\right),\left(\text{0.5,0.4,0.4}\right))$	$\:(\left(\text{0.2,0.1}\right),\left(\text{0.7,0.8}\right))$
FM2	$\:(\left(\text{0.9,0.6}\right),\left(\text{0.2,0.2}\right))$	$\:(\left(\text{0.6,0.2}\right),\left(\text{0.3,0.7,0.5}\right))$	$\:(\left(\text{0.2,0.2}\right),\left(\text{0.9,0.2}\right))$
FM3	$\:(\left(\text{0.3,0.4,0.1}\right),\left(\text{0.3,0.8}\right))$	$\:(\left(\text{0.1,0.3,0.6}\right),\left(\text{0.1,0.4,0.3}\right))$	$\:(\left(\text{0.7,0.5,0.5}\right),\left(\text{0.2,0.6,0.2}\right))$
FM4	$\:(\left(\text{0.4,0.5,0.8}\right),\left(\text{0.6,0.2}\right))$	$\:(\left(\text{0.4,0.5}\right),\left(\text{0.7,0.2}\right))$	$\:(\left(\text{0.6,0.6,0.5}\right),\left(\text{0.2,0.6}\right))$
FM5	$\:(\left(\text{0.5,0.9,0.4}\right),\left(\text{0.2,0.1,0.1}\right))$	$\:(\left(\text{0.7,0.4}\right),\left(\text{0.8,0.5,0.6}\right))$	$\:(\left(\text{0.1,0.6,0.2}\right),\left(\text{0.1,0.2}\right))$
FM6	$\:(\left(\text{0.4,0.9}\right),\left(\text{0.3,0.5}\right))$	$\:(\left(\text{0.9,0.4}\right),\left(\text{0.3,0.2,0.2}\right))$	$\:(\left(\text{0.7,0.4,0.8}\right),\left(\text{0.4,0.1}\right))$
FM7	$\:(\left(\text{0.7,0.3}\right),\left(\text{0.4,0.7,0.7}\right))$	$\:(\left(\text{0.3,0.2}\right),\left(\text{0.1,0.1,0.8}\right))$	$\:(\left(\text{0.1,0.8}\right),\left(\text{0.7,0.2}\right))$
FM8	$\:(\left(\text{0.8,0.1,0.5}\right),\left(\text{0.2,0.1,0.6}\right))$	$\:(\left(\text{0.2,0.7,0.2}\right),\left(\text{0.8,0.3,0.1}\right))$	$\:(\left(\text{0.6,0.1,0.5}\right),\left(\text{0.1,0.6,0.5}\right))$
FM9	$\:(\left(\text{0.6,0.8}\right),\left(\text{0.4,0.3,0.6}\right))$	$\:(\left(\text{0.6,0.2}\right),\left(\text{0.2,0.1}\right))$	$\:(\left(\text{0.8,0.2}\right),\left(\text{0.6,0.5}\right))$
FM10	$\:(\left(\text{0.6,0.4,0.6}\right),\left(\text{0.7,0.7}\right))$	$\:(\left(\text{0.4,0.5}\right),\left(\text{0.2,0.6,0.2}\right))$	$\:(\left(\text{0.2,0.2}\right),\left(\text{0.3,0.7}\right))$
	DM3		
FM1	$\:(\left(\text{0.1,0.6,0.1}\right),\left(\text{0.5,0.8}\right))$	$\:(\left(\text{0.3,0.7}\right),\left(\text{0.8,0.6}\right))$	$\:(\left(\text{0.1,0.8,0.6}\right),\left(\text{0.4,0.7}\right))$
FM2	$\:(\left(\text{0.4,0.2,0.7}\right),\left(\text{0.5,0.5,0.3}\right))$	$\:(\left(\text{0.3,0.3}\right),\left(\text{0.2,0.4,0.9}\right))$	$\:(\left(\text{0.4,0.1}\right),\left(\text{0.1,0.2,0.8}\right))$
FM3	$\:(\left(\text{0.8,0.1}\right),\left(\text{0.4,0.7}\right))$	$\:(\left(\text{0.6,0.5}\right),\left(\text{0.6,0.9,0.5}\right))$	$\:(\left(\text{0.4,0.4}\right),\left(\text{0.5,0.9}\right))$
FM4	$\:(\left(\text{0.7,0.7,0.4}\right),\left(\text{0.1,0.1}\right))$	$\:(\left(\text{0.1,0.1,0.2}\right),\left(\text{0.8,0.9,0.6}\right))$	$\:(\left(\text{0.7,0.6}\right),\left(\text{0.3,0.7,0.7}\right))$
FM5	$\:(\left(\text{0.8,0.9,0.6}\right),\left(\text{0.1,0.4}\right))$	$\:(\left(\text{0.2,0.4,0.1}\right),\left(\text{0.5,0.8}\right))$	$\:(\left(\text{0.6,0.6}\right),\left(\text{0.1,0.7,0.7}\right))$
FM6	$\:(\left(\text{0.6,0.5}\right),\left(\text{0.7,0.8}\right))$	$\:(\left(\text{0.5,0.3,0.3}\right),\left(\text{0.7,0.1,0.4}\right))$	$\:(\left(\text{0.5,0.7,0.2}\right),\left(\text{0.6,0.8}\right))$
FM7	$\:(\left(\text{0.2,0.5,0.8}\right),\left(\text{0.2,0.7}\right))$	$\:(\left(\text{0.8,0.8,0.1}\right),\left(\text{0.6,0.4,0.7}\right))$	$\:(\left(\text{0.6,0.2,0.3}\right),\left(\text{0.8,0.2,0.7}\right))$
FM8	$\:(\left(\text{0.8,0.3}\right),\left(\text{0.7,0.6}\right))$	$\:(\left(\text{0.5,0.1,0.6}\right),\left(\text{0.4,0.2}\right))$	$\:(\left(\text{0.5,0.7}\right),\left(\text{0.2,0.7}\right))$
FM9	$\:(\left(\text{0.5,0.6,0.1}\right),\left(\text{0.8,0.2}\right))$	$\:(\left(\text{0.8,0.3,0.4}\right),\left(\text{0.5,0.1}\right))$	$\:(\left(\text{0.7,0.9,0.8}\right),\left(\text{0.5,0.6}\right))$
FM10	$\:(\left(\text{0.4,0.1}\right),\left(\text{0.8,0.3,0.8}\right))$	$\:(\left(\text{0.1,0.1}\right),\left(\text{0.7,0.4}\right))$	$\:(\left(\text{0.2,0.7,0.4}\right),\left(\text{0.5,0.5,0.1}\right))$

Step 2: The weights of the decision makers are calculated using the TOPSIS method with Eqs. (13)–(18) and the resulting weight vector is $\:w=\left[0.3250\:0.3367\:0.3382\right]$.

Step 3: The prospect decision matrices $\:{R}^{k}(k=\text{1,2},3)$ are constructed based on prospect theory using (21) and are presented in Table 3.

**Table 3:** Prospect decision matrices of each expert.

Failure modes	$\:{{R}}^{1}$			$\:{{R}}^{2}$			$\:{{R}}^{3}$		
	O	S	D	O	S	D	O	S	D
FM1	$\:0.0389$	$\:0.0331$	$\:0.0534$	$\:0.0573$	$\:0.\:0421$	$\:0.0124$	$\:0.0404$	$\:0.0325$	$\:0.0525$
FM2	$\:0.0317$	$\:0.0480$	$\:0.0234$	$\:0.0693$	$\:0.\:0288$	$\:0.0317$	$\:0.0321$	$\:0.0488$	$\:0.0241$
FM3	$\:0.0393$	$\:0.0523$	$\:0.0229$	$\:0.0184$	$\:0.\:0385$	$\:0.0580$	$\:0.0396$	$\:0.0527$	$\:0.0244$
FM4	$\:0.0158$	$\:0.0188$	$\:0.0478$	$\:0.\:0399$	$\:0.\:0393$	$\:0.0574$	$\:0.0153$	$\:0.0185$	$\:0.0469$
FM5	$\:0.0412$	$\:0.0278$	$\:0.0684$	$\:0.\:0631$	$\:0.\:0430$	$\:0.0600$	$\:0.0348$	$\:0.0278$	$\:0.0677$
FM6	$\:0.0493$	$\:0.0413$	$\:0.0483$	$\:0.\:0457$	$\:0.\:0638$	$\:0.0613$	$\:0.0518$	$\:0.0415$	$\:0.0497$
FM7	$\:0.0590$	$\:0.0647$	$\:0.0599$	$\:0.\:0273$	$\:0.\:0285$	$\:0.0399$	$\:0.0573$	$\:0.0653$	$\:0.0617$
FM8	$\:0.0489$	$\:0.0517$	$\:0.0354$	$\:0.\:0350$	$\:0.\:0328$	$\:0.0396$	$\:0.0494$	$\:0.0524$	$\:0.0346$
FM9	$\:0.0572$	$\:0.0301$	$\:0.0300$	$\:0.\:0504$	$\:0.\:0538$	$\:0.0436$	$\:0.0582$	$\:0.0297$	$\:0.0276$
FM10	$\:0.0433$	$\:0.0393$	$\:0.0429$	$\:0.\:0286$	$\:0.\:0414$	$\:0.0272$	$\:0.0461$	$\:0.0391$	$\:0.0424$

Step 4: The weights of risk factors $\:{w}_{j}^{k}$ $\:\left(j=\text{1,2},3;k=\text{1,2},3\right)$ for each expert were calculated using (20) and are given in Table 4.

**Table 4:** Weights of risk factors for each DMs.

	$\:{{R}}^{1}$			$\:{{R}}^{2}$			$\:{{R}}^{3}$		
	O	S	D	O	S	D	0	S	D
W	$\:0.5833$	$\:0.3055$	$\:0.1111$	$\:0.5909$	$\:0.\:03182$	$\:0.0909$	$\:0.2200$	$\:0.6800$	$\:0.1000$

Step 5: The weighted decision matrix $\:{V}^{k}$ $\:\left(k=\text{1,2},3\right)$ for each expert is computed using (27) and is provided in Table 5.

**Table 5:** Weighted decision matrix.

Failure modes	$\:{{V}}^{1}$			$\:{{V}}^{2}$			$\:{{V}}^{3}$		
	O	S	D	O	S	D	O	S	D
FM1	$\:0.0227$	$\:0.0101$	$\:0.0059$	$\:0.0339$	$\:0.\:0134$	$\:0.\:0011$	$\:0.\:0089$	$\:0.\:0221$	$\:0.\:0053$
FM2	$\:0.0185$	$\:0.0147$	$\:0.0026$	$\:0.\:0409$	$\:0.\:0092$	$\:0.\:0029$	$\:0.\:0071$	$\:0.\:0332$	$\:0.\:0024$
FM3	$\:0.0229$	$\:0.0160$	$\:0.0025$	$\:0.\:0109$	$\:0.\:0122$	$\:0.\:0053$	$\:0.\:0087$	$\:0.\:0358$	$\:0.\:0024$
FM4	$\:0.0092$	$\:0.0057$	$\:0.0053$	$\:0.\:0236$	$\:0.\:0125$	$\:0.\:0052$	$\:0.\:0034$	$\:0.\:0126$	$\:0.\:0047$
FM5	$\:0.0240$	$\:0.0085$	$\:0.0076$	$\:0.\:0373$	$\:0.\:0137$	$\:0.\:0055$	$\:0.\:0077$	$\:0.\:0189$	$\:0.\:0068$
FM6	$\:0.0288$	$\:0.0126$	$\:0.0054$	$\:0.\:0270$	$\:0.\:0203$	$\:0.\:0056$	$\:0.\:0114$	$\:0.\:0282$	$\:0.\:0050$
FM7	$\:0.0344$	$\:0.0198$	$\:0.0067$	$\:0.\:0161$	$\:0.\:0091$	$\:0.\:0036$	$\:0.\:0126$	$\:0.\:0444$	$\:0.\:0062$
FM8	$\:0.0286$	$\:0.0158$	$\:0.0039$	$\:0.\:0207$	$\:0.\:0104$	$\:0.\:0036$	$\:0.\:0109$	$\:0.\:0357$	$\:0.\:0035$
FM9	$\:0.0334$	$\:0.0092$	$\:0.0033$	$\:0.\:0298$	$\:0.\:0171$	$\:0.\:0040$	$\:0.\:0128$	$\:0.\:0202$	$\:0.\:0028$
FM10	$\:0.0252$	$\:0.0120$	$\:0.0048$	$\:0.\:0169$	$\:0.\:0132$	$\:0.\:0025$	$\:0.\:0101$	$\:0.\:0266$	$\:0.\:0042$

Step 6: The aggregated weighted decision matrix $\:V=\left({t}_{ij}\right),\:i=\text{1,2},\dots\:,10;j=\text{1,2},3$ is calculated using (28), and the results are listed in Table 6.

**Table 6:** Aggregated weighted decision matrix.

Failure modes	$\:{V}$		
	O	S	D
FM1	$\:0.0218$	$\:0.0153$	$\:0.0041$
FM2	$\:0.0223$	$\:0.0191$	$\:0.0026$
FM3	$\:0.0141$	$\:0.0215$	$\:0.0034$
FM4	$\:0.0121$	$\:0.0103$	$\:0.0051$
FM5	$\:0.0230$	$\:0.0138$	$\:0.0066$
FM6	$\:0.0223$	$\:0.0205$	$\:0.0053$
FM7	$\:0.0209$	$\:0.0246$	$\:0.0055$
FM8	$\:0.0199$	$\:0.0208$	$\:0.0037$
FM9	$\:0.0252$	$\:0.0156$	$\:0.0033$
FM10	$\:0.0173$	$\:0.0174$	$\:0.0038$

Step 7: The border approximation area matrix $\:B=\left({b}_{j}\right),\:j=\text{1,2},3$ is calculated using (29) as $\:B=\left[0.0195\:\:\:0.0174\:\:\:0.0042\right]$.

Step 8: The distance matrix $\:D=\left({d}_{ij}\right)$ for $\:i=\text{1,2},\dots\:,10$; $\:j=\text{1,2},3$ is computed using (30) as shown in Table 7.

**Table 7:** Distance matrix.

Failure modes	Distances		
	O	S	D
FM1	$\:0.00236$	$\:-0.00209$	$\:-0.00011$
FM2	$\:0.00282$	$\:0.00173$	$\:-0.00156$
FM3	$\:-0.00538$	$\:0.00408$	$\:-0.00076$
FM4	$\:-0.00735$	$\:-0.00705$	$\:0.00088$
FM5	$\:0.00357$	$\:-0.00363$	$\:0.00241$
FM6	$\:0.00286$	$\:0.00310$	$\:0.00111$
FM7	$\:0.00147$	$\:0.00722$	$\:0.00013$
FM8	$\:0.00048$	$\:0.00337$	$\:-0.00053$
FM9	$\:0.00578$	$\:-0.00181$	$\:-0.00084$
FM10	$\:-0.00212$	$\:-0.00004$	$\:-0.00037$

Step 9: The final $\:{S}_{i}$ values for $\:i=\text{1,2},\dots\:,10$ are determined by using (31) as given in Table 8.

**Table 8:** S values.

	FM1	FM2	FM3	FM4	FM5	FM6	FM7	FM8	FM9	FM10
S	$\:0.0002$	$\:0.0030$	$\:-0.0021$	$\:-0.0135$	$\:0.0024$	$\:0.0071$	$\:0.0100$	$\:0.0033$	$\:0.0031$	$\:-0.0025$

Step 10: According to results, the risk priority ranking of ten failure modes is found to be

$$\:FM7>FM6>FM8>FM9>FM2>FM5>FM1>FM3>FM10>FM4.$$

**Fig. 2:**
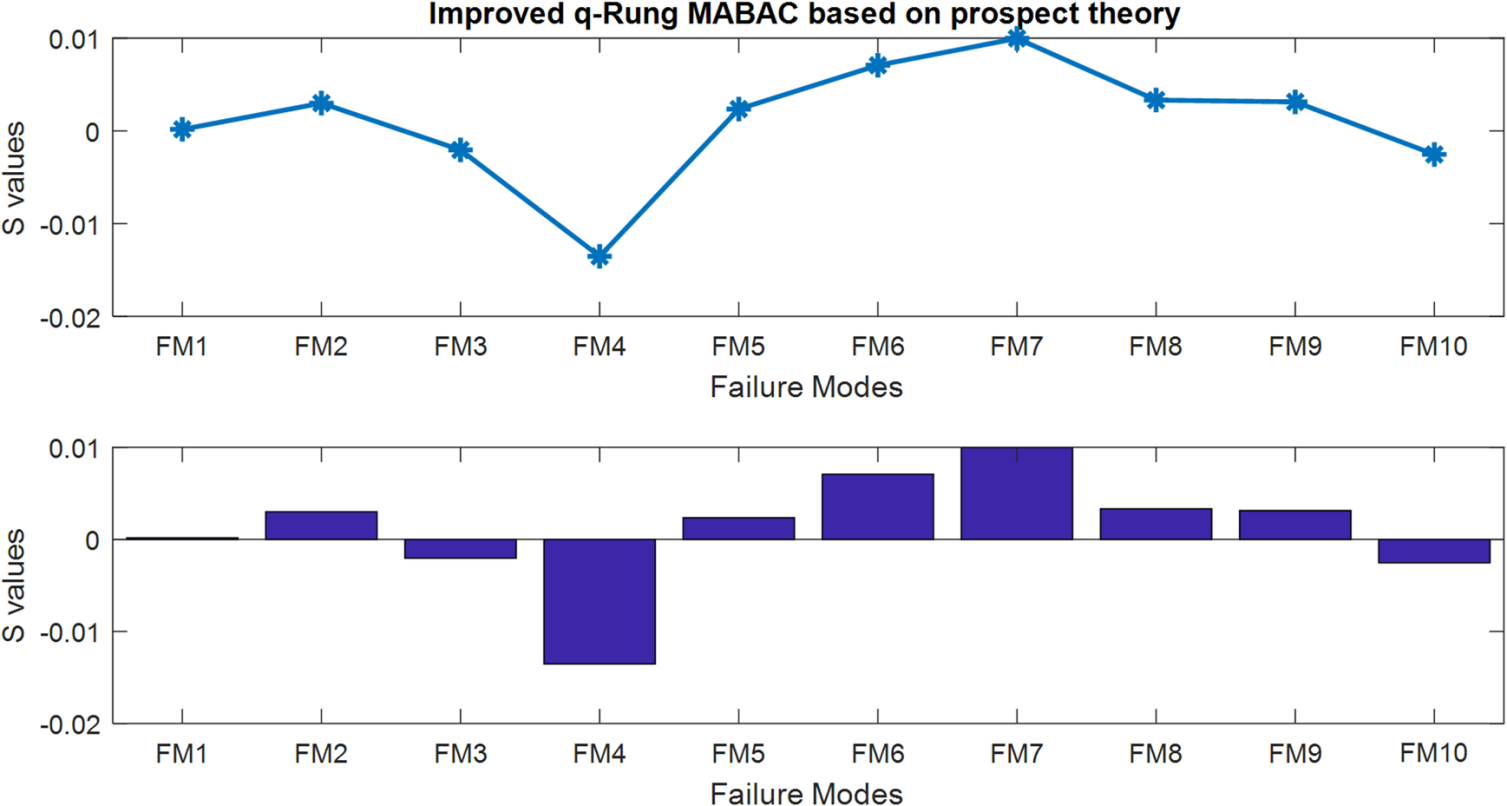
Risk graphs of each failure mode.

It is concluded that FM7 has the highest risk priority. The line and bar charts illustrating the risk evaluations of each failure mode are provided in Fig. 2.

The originality of the proposed method lies in its systematic integration of prospect theory into the FMEA framework under the q-rung orthopair hesitant fuzzy environment. Unlike traditional FMEA methodologies, which typically aggregate expert judgments directly without accounting for cognitive biases or varying psychological reference points, this approach incorporates the value and probability weighting functions of prospect theory individually into each decision matrix constructed by the experts. By doing so, it captures the nonlinear perception of gains and losses, as well as the decision makers’ attitude toward risk—particularly in the presence of uncertainty and hesitation represented by q-ROHFNs. This integration enables the model to reflect more realistic human decision behaviour and reduces the loss of real-world information. As a result, the proposed model yields more robust, behaviourally consistent, and accurate prioritizations of failure modes, thereby enhancing the reliability and interpretability of the FMEA process.

### Sensitivity analysis

Since the proposed method introduces a novel risk evaluation approach based on prospect theory under a q-rung orthopair fuzzy environment, it is essential to examine how the variations in key input parameters influence the final outcomes. Sensitivity analysis is conducted under three distinct scenarios, while the results of the original numerical example are referred to as Scenario 0 for baseline comparison. The final $\:{S}_{i}$​ values obtained under each scenario are summarized in Table 9 and the ranking of failure modes in each scenario is given in Table 10.

**Table 9:** S values of failure modes for each scenario.

	Scenario 0	Scenario 1	Scenario 2	Scenario 3
	$\:\lambda\:=\left[0.3250\:0.3367\:0.3382\right]$ $\:\alpha\:=0.88;\beta\:=0.88;\theta\:=2.25;$ $\:\gamma\:=0.61;\delta\:=0.72$ $\:{w}^{1}=\left[0.5833\:0.3055\:0.1111\right]$ $\:{w}^{2}=\left[0.5909\:0.3182\:0.0909\right]$ $\:{w}^{3}=\left[0.2200\:0.6800\:0.1000\right]$	$\:\lambda\:=\left[0.3\:0.4\:0.3\right]$	$\:\alpha\:=0.5;$ $\:\beta\:=0.5;$ $\:\theta\:=5;$ $\:\gamma\:=0.5;$ $\:\delta\:=0.5$	$\:{w}^{1}=\left[0.5\:0.3\:0.2\right]$ $\:{w}^{2}=\left[0.6\:0.3\:0.1\right]$ $\:{w}^{3}=\left[0.2\:0.6\:0.2\right]$
FM1	$\:0.002$	$\:0.0007$	$\:0.0002$	$\:0.0011$
FM2	$\:0.0030$	$\:0.0037$	$\:0.0029$	$\:0.0021$
FM3	$\:-0.0021$	$\:-0.0032$	$\:-0.0031$	$\:-0.0035$
FM4	$\:-0.0135$	$\:-0.0123$	$\:-0.0181$	$\:-0.0116$
FM5	$\:0.0024$	$\:0.0035$	$\:0.0021$	$\:0.0046$
FM6	$\:0.0071$	$\:0.0074$	$\:0.0084$	$\:0.0072$
FM7	$\:0.0100$	$\:0.0077$	$\:0.0104$	$\:0.0099$
FM8	$\:0.0033$	$\:0.0023$	$\:0.0041$	$\:0.0023$
FM9	$\:0.0031$	$\:0.0037$	$\:0.0034$	$\:0.0020$
FM10	$\:-0.0025$	$\:-0.0032$	$\:-0.0026$	$\:-0.0026$

In Scenario 1, the sensitivity of the method to changes in decision-makers’ weights is investigated. While in Scenario 0 the weights were derived using the TOPSIS method (resulting in $\:\lambda\:=\left[\text{0.3250,0.3367,0.3382}\right])$, in Scenario 1 they are manually assigned as $\:\lambda\:=\left[\text{0.3,0.4,0.3}\right]$. Table 9 illustrates that even slight adjustments in DMs’ importance levels change the risk prioritization results, highlighting the model’s responsiveness to weighting schemes.

In Scenario 2, the parameters of the prospect theory model—specifically the coefficients for the value and probability weighting functions—are modified. While Scenario 0 employs $\:(\alpha\:,\beta\:,\theta\:,\gamma\:,\delta\:)=\left(\text{0.88,0.88,2.25,0.61,0.72}\right)$, Scenario 2 uses a more risk-averse configuration: $\:(\alpha\:,\beta\:,\theta\:,\gamma\:,\delta\:)=(\text{0.5,0.5,5},\text{0.5,0.5})$. These adjustments represent DMs who perceive losses more strongly and distort probabilities more severely. As seen in Table 9 these changes also result in modified rankings.

In Scenario 3, the weights of risk factors—Occurrence (O), Severity (S), and Detection (D) assigned by each DM are slightly altered from their original values in Scenario 0. Although the changes are minor and remain consistent with expert expectations, the resulting rank order of failure modes differs, confirming the method’s sensitivity to expert judgments at the factor level.

**Table 10:** Ranking of failure modes for each scenario.

Scenarios	Rankings
Scenarios$\:\:0$	$\:FM7>FM6>FM8>FM9>FM2>FM5>FM1>FM3>FM10>FM4$
Scenarios$\:\:1$	$\:FM7>FM6>FM9>FM2>FM5>FM8>FM1>FM3>FM10>FM4$
Scenarios$\:\:2$	$\:FM7>FM6>FM8>FM9>FM2>FM5>FM1>FM10>FM3>FM4$
Scenarios $\:3$	$\:FM7>FM6>FM5>FM8>FM2>FM9>FM1>FM10>FM3>FM4$

**Fig. 3:**
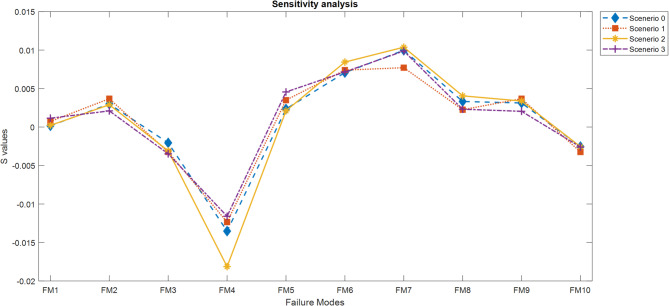
S values of failure modes for each scenario.

The graphical comparison of the $\:{S}_{i}$​ ​ values across all four scenarios is illustrated in Fig. 3. Each line corresponds to a scenario and clearly depicts the variation in evaluations across different parameter settings. Notably, FM7 consistently emerges as the top-priority failure mode, demonstrating a high level of robustness in the proposed approach.

**Fig. 4:**
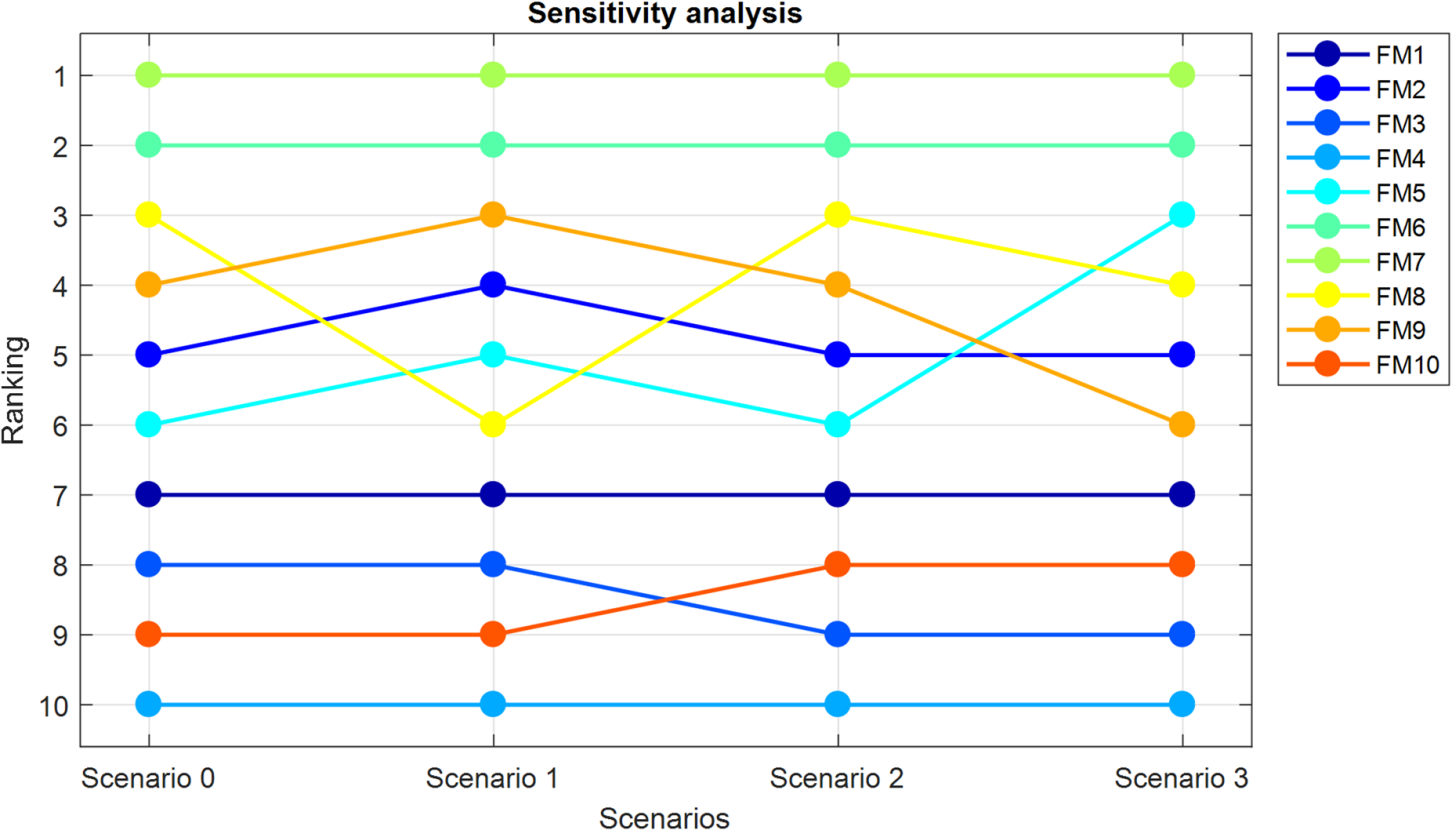
Rankings of failure modes for each scenario.

It can be concluded from the results given in the tables and figures that the small changes in parameters have a considerable effect on the results, as expected. The results present that the proposed method is sensitive to given parameters (Fig. 4). Since the main idea of the prospect theory is the influence of human behaviours on decisions and the risk in cases is highly dependent on human behaviours, the proposed method is very useful in risk evaluations.

The sensitivity analysis confirms that even moderate changes in DM weights, prospect theory parameters, or risk factor weightings can significantly affect the final risk rankings. This responsiveness is an expected outcome, considering that the proposed method is designed to involve the human-centric uncertainty and psychological behaviour embedded in prospect theory. The results validate the model’s practical relevance and adaptability for expert-driven, behaviour-sensitive decision-making contexts such as FMEA.

### Comparative discussion

#### Method-based comparison

**Fig. 5:**
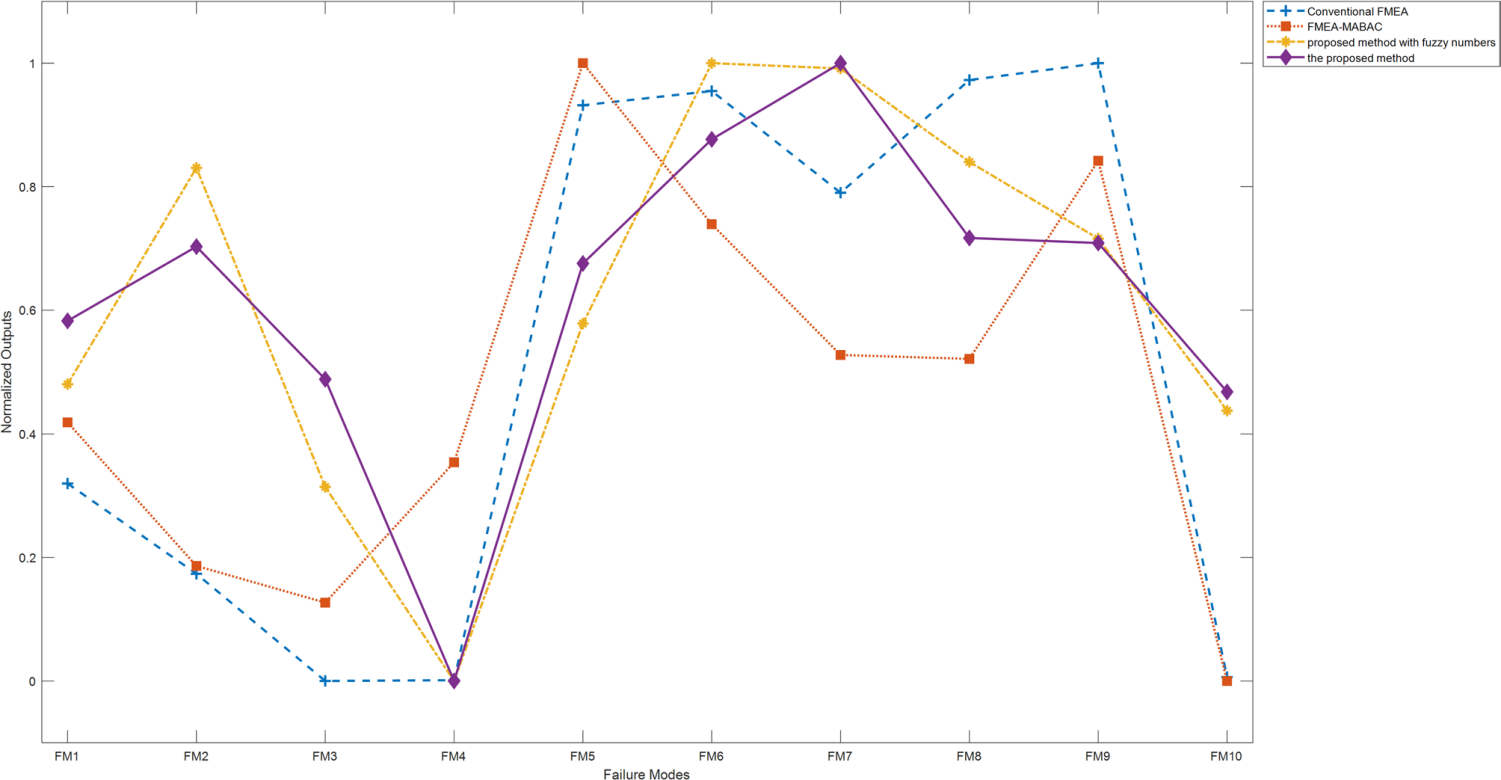
Comparison of rankings with normalized values.

To confirm the effectiveness and the potency of the proposed method, a comparison analysis is conducted by the conventional FMEA method (M1), FMEA with MABAC method (M2), the proposed method with fuzzy numbers instead of q-rung orthopair hesitant fuzzy numbers (M3). The proposed method using q-ROHFNs and prospect theory is referred to as (M4). The final results of comparison are presented in Table 11 and visualized in Fig. 5.

For consistency, the conventional FMEA and FMEA-MABAC methods are executed using q-ROHFNs. The reason behind this is the novelty of integrating q-ROHFNs into the FMEA framework, as this is the first instance of such an application in the literature. In addition, the influence of q-ROHFNs on the evaluation process is demonstrated by transforming the decision matrices into classical fuzzy numbers and comparing the outcomes with M4.

**Table 11:** Results of the comparisons.

Failure modes	M1		M2		M3		M4 (proposed)	
	$\:{S}_{i}$	Ranking	$\:{S}_{i}$	Ranking	$\:{S}_{i}$	Ranking	$\:{S}_{i}$	Ranking
FM1	$\:-0.6387$	6	$\:0.5580$	6	$\:0.4800$	7	$\:0.002$	7
FM2	$\:-0.6704$	7	$\:0.4930$	8	$\:0.8305$	4	$\:0.0030$	5
FM3	$\:-0.7081$	10	$\:0.4764$	9	$\:0.3142$	9	$\:-0.0021$	8
FM4	$\:-0.7078$	9	$\:0.5399$	7	$\:0$	10	$\:-0.0135$	10
FM5	$\:-0.5059$	4	$\:0.7205$	1	$\:0.5783$	6	$\:0.0024$	6
FM6	$\:-0.5009$	3	$\:0.6476$	3	$\:1.0000$	1	$\:0.0071$	2
FM7	$\:-0.5366$	5	$\:0.5884$	4	$\:0.9913$	2	$\:0.0100$	1
FM8	$\:-0.4970$	2	$\:0.5866$	5	$\:0.8400$	3	$\:0.0033$	3
FM9	$\:-0.4911$	1	$\:0.6763$	2	$\:0.7158$	5	$\:0.0031$	4
FM10	$\:-0.7067$	8	$\:0.4409$	10	$\:0.4377$	8	$\:-0.0025$	9

The first comparison is the classical FMEA method. In the classical FMEA method, the aggregated decision matrices of all experts were computed using the q-ROHFWA operator, and the final risk evaluations were obtained using a score function applied to the aggregated values. Due to the nature of the score function, all final values in this method are negative. In this scenario, FM9 was identified as the most critical failure mode. However, this approach does not consider interdependencies between criteria or DMs’ behavioural aspects.

The second comparison is made with the FMEA MABAC method. This variant applies only the MABAC ranking process to the conventional aggregated matrix without incorporating the prospect theory. FM5 emerged as the most critical failure mode in this scenario, pushing FM9 to the second place. This shift illustrates the influence of a MCDM technique (MABAC) on the prioritization outcomes, even without incorporating behavioural models.

The third comparison is conducted with the fuzzy numbers. To benchmark the proposed methodology against more classical fuzzy systems, the q-ROHFNs in the decision matrices were reduced to standard fuzzy numbers using the method suggested by^48^.While the underlying prospect theory was retained, this conversion enabled a comparison with the approach followed by Yazdi et al. (2017). FM6 ranked first in this configuration. The differences in rankings particularly when compared to M4 underscore the power and expressiveness of q-ROHFNs in capturing complex expert hesitancies and uncertainties.

**Fig. 6:**
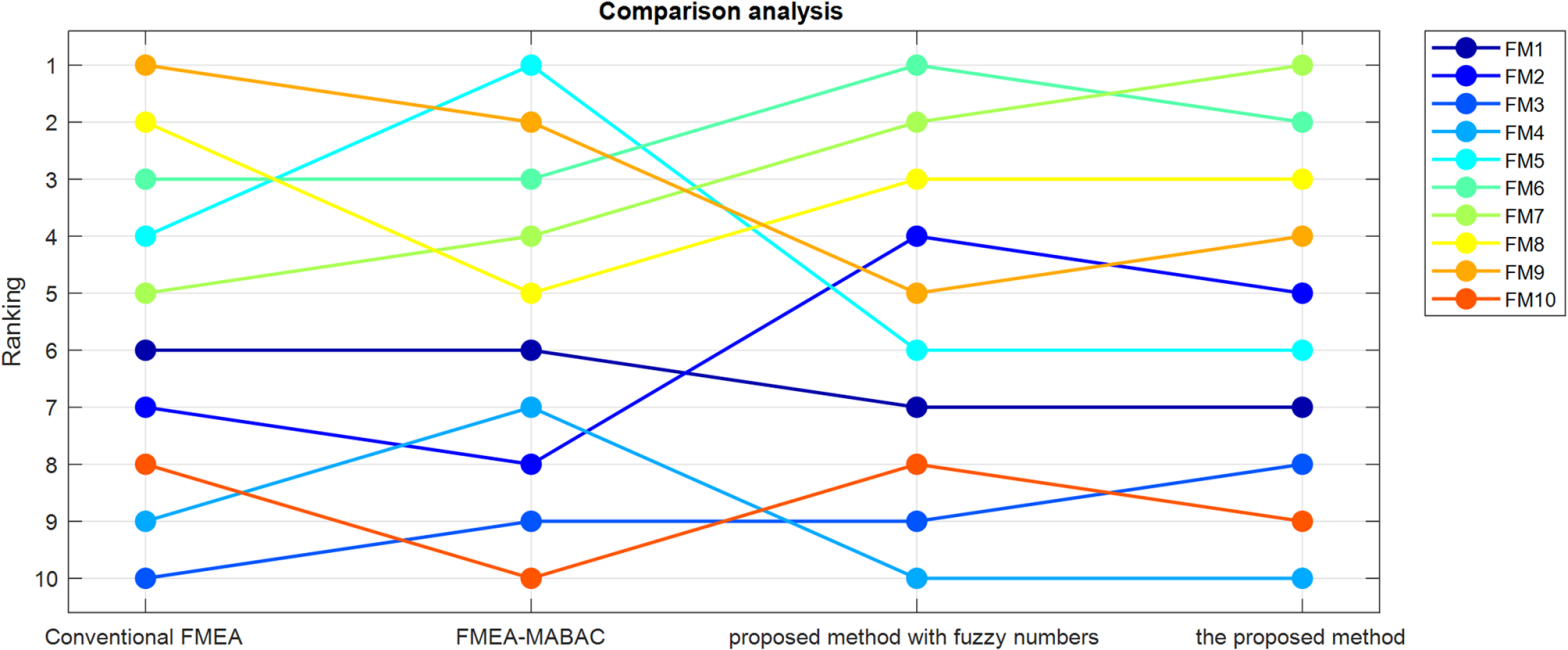
Comparison of rankings with normalized values.

To further highlight the differences in prioritization, the failure mode rankings for each method are summarized in Table 12 and illustrated in Fig. 6.

**Table 12:** Ranking comparison of the methods.

	Rankings
M1	$\:FM9>FM8>FM6>FM5>FM7>FM1>FM2>FM10>FM4>FM3$
M2	$\:FM5>FM9>FM6>FM7>FM8>FM1>FM4>FM2>FM3>FM10$
M3	$\:FM6>FM7>FM8>FM2>FM9>FM5>FM1>FM10>FM3>FM4$
M4	$\:FM7>FM6>FM8>FM9>FM2>FM5>FM1>FM3>FM10>FM4$

The comparative analysis clearly demonstrates the superiority of the proposed method (M4) over the classical FMEA (M1), the FMEA-MABAC method (M2), and the fuzzy-based version of the proposed method (M3). These findings provide valuable insights into the benefits and implications of incorporating both q-rung orthopair hesitant fuzzy numbers (q-ROHFNs) and prospect theory into FMEA framework. One of the most significant distinctions of the proposed method lies in its integration of human behavioural aspects through prospect theory at an early stage of the decision-making process. While traditional FMEA methods such as M1 and M2 assume that decision makers act rationally and consistently, the proposed method explicitly accounts for psychological biases such as loss aversion, risk preference asymmetry, and probability distortion, which are often observed in real-world risk assessments. This feature enables more realistic modelling of expert evaluations, particularly in high-stakes or uncertain environments.

In terms of mathematical representation of uncertainty, the use of q-ROHFNs allows the method to capture a richer spectrum of expert hesitancy compared to traditional fuzzy numbers. While fuzzy-based models (as in M3) can express uncertainty to a certain degree, they are limited in distinguishing multiple degrees of membership and non-membership simultaneously. The q-ROHFN framework, on the other hand, allows decision makers to express multiple and possibly conflicting hesitant values, which are then systematically integrated into the model. This leads to a more comprehensive and nuanced reflection of human judgment, especially when there is disagreement or imprecision in expert opinions. Furthermore, the use of the Best-Worst Method for criteria weighting with MABAC for ranking provides a robust multicriteria decision-making foundation. Compared to the classical aggregation used in M1 or the conventional MABAC structure in M2, this integrated approach enables the model to effectively balance subjectivity in criteria importance with objectivity in alternative performance.

#### Distance-based comparison

In this section, a distance-based comparison is conducted to evaluate the effectiveness and consistency of the proposed model against existing decision-making frameworks. Since the MABAC method operates based on distance computations from an ideal solution, analysing the divergence in rankings and scores under various distance measures provides critical insight into the model’s sensitivity and reliability. Particularly, the inclusion of a generalized q-ROHF Minkowski distance given in (9) in this study allows the decision-makers to flexibly tune the distance behaviour through the selection of $\:\delta\:$ parameters, encompassing commonly used distances such as Hamming $\:(\delta\:=1)$ and Euclidean $\:(\delta\:=2)$ as special cases. The comparative results presented here aim to highlight how different distance functions influence the final risk prioritization and demonstrate the robustness of the proposed method in capturing expert hesitation and uncertainty under a flexible distance structure.

Recall that the conventional FMEA method is M1, FMEA with MABAC method is M2, the proposed method with fuzzy numbers instead of q-rung orthopair hesitant fuzzy numbers is M3 and the proposed method using q-ROHFNs and prospect theory is referred to as M4. To see the effect of adjustable distance we take $\:\delta\:=1$ (Hamming), $\:\delta\:=2$ (Euclidean) and $\:\delta\:=10$ and recalculate the compared methods.

**Table 13:** Distance-based ranking comparison of the methods.

		Rankings
M1	$\:\delta\:=1$	$\:FM9>FM8>FM6>FM5>FM7>FM1>FM2>FM10>FM4>FM3$
	$\:\delta\:=2$	$\:FM9>FM8>FM6>FM5>FM7>FM1>FM2>FM10>FM4>FM3$
	$\:\delta\:=9$	$\:FM9>FM8>FM6>FM5>FM7>FM1>FM2>FM10>FM4>FM3$
M2	$\:\delta\:=1$	$\:FM5>FM9>FM6>FM8>FM7>FM4>FM1>FM2>FM3>FM10$
	$\:\delta\:=2$	$\:FM5>FM9>FM6>\varvec{F}\varvec{M}7>\varvec{F}\varvec{M}8>\varvec{F}\varvec{M}1>F\varvec{M}4>FM2>FM3>FM10$
	$\:\delta\:=9$	$\:FM5>FM9>FM6>FM7>\varvec{F}\varvec{M}1>\varvec{F}\varvec{M}8>FM4>FM2>FM3>FM10$
M3	$\:\delta\:=1$	$\:FM6>FM7>FM8>FM2>FM9>FM5>FM1>FM10>FM3>FM4$
	$\:\delta\:=2$	$\:FM6>FM7>FM8>FM2>FM9>FM5>FM1>FM10>FM3>FM4$
	$\:\delta\:=9$	$\:FM6>FM7>FM8>FM2>FM9>FM5>FM1>FM10>FM3>FM4$
M4	$\:\delta\:=1$	$\:FM7>FM6>FM8>FM2>FM9>FM5>FM1>FM10>FM3>FM4$
	$\:\delta\:=2$	$\:FM7>FM6>FM8>\varvec{F}\varvec{M}9>\varvec{F}\varvec{M}2>FM5>FM1>\varvec{F}\varvec{M}3>\varvec{F}\varvec{M}10>FM4$
	$\:\delta\:=9$	$\:FM7>FM6>\varvec{F}\varvec{M}9>\varvec{F}\varvec{M}8>\varvec{F}\varvec{M}5>\varvec{F}\varvec{M}1>\varvec{F}\varvec{M}2>FM3>FM10>FM4$

As can be seen in Table 13, Since conventional FMEA method (M1) has no distance, no change was the expected result. In the FMEA with MABAC (M2), the top failure modes remain consistent (FM5 > FM9 > FM6), but minor shifts occur in the middle and lower rankings. For example, FM8 moves between 4th and 7th positions depending on $\:\delta\:$. This reflects moderate sensitivity to the distance parameter and highlights the flexibility of the model in capturing such variation. If we use only fuzzy numbers (M3), the rankings are identical across all $\:\delta\:$ values. The fixed ordering implies that the Minkowski parameter has limited influence on this fuzzy number pattern. However, in the proposed method using q-ROHFNs and prospect theory (M4) notable changes occur. While the top two rankings (FM7, FM6) remain stable, other failure modes exhibit ranking swaps based on $\:\delta\:$. This suggests that the proposed method’s assessments are more sensitive to distance metrics and may contain more nuanced hesitation or inconsistency, which the proposed model successfully captures.

These results demonstrate that the proposed Minkowski distance formulation enables the method to adapt its ranking behaviour depending on the selected δ parameter. This flexibility allows decision-makers to fine-tune the sensitivity of the model, which is particularly beneficial in situations where expert judgments vary significantly in granularity or reliability. Overall, the distance-based analysis reinforces the robustness and adaptability of the proposed method and aligning well with the goal of modelling human judgment under uncertainty.

## Conclusion and future study

Since risk management can play a key role for many systems, it may be strategic and vital to carry out in a more controlled and planned manner. With this mind, measures to be taken to detect and prevent any danger that may occur during the flight landing is of vital importance. Therefore, this problem is handled as an FMEA problem and failure modes with risk factors are determined. Further, the system is supported by MABAC method in order to take risk management to the next level by eliminating the disadvantages in classical FMEA. In addition, classic MABAC method has been strengthened by including human psychology in the process, and psychological behaviour has been processed in a way that can be handled separately for each person in the process using with prospect theory. Also, the fact that probability information, which is one of the components of the prospect theory, is not used in decision problems is a deficiency in the literature, and it is aimed to obtain more sensitive results by including the probability density function in the system in order to use the theory in a way that suits its purpose. TOPSIS arguments, which is a consistent decision-making method, are used in order to objectively calculate the decision maker weights and to examine the sensitive effect on the results. It has been observed that even with a very small change, the results and rankings change. Similarly, best-worst method, in which we can consider the relationship between risk factors, is preferred to determine the weights of risk factors that may have a significant effect on the results. Apart from these, the process has been advanced in the q-rung orthopair hesitant fuzzy environment, which can model uncertainty in a wider and more comprehensive area. Finally, the key conclusions of the study can be summarized as follows:

- Unlike many other methods, applying the prospect theory to each decision maker’s choices rather than an aggregated decision matrix yields more effective results. The change in risk rankings is an example of how powerful and essential the method is.
- The proposed model can give sensitive results according to changes in parameters, which can be considered human behaviours with the effect of prospect theory. This adaptable structure of the method brings advantages and convenience in real-life applications.
- When individual attitude, probability information, the MABAC method and the FMEA method come together, the proposed method promises significant results in risk assessment with the advantage of q-rung orthopair hesitant information.

However, despite its advantages, the proposed method still has limitations. The computational process becomes increasingly complex when the number of decision makers and failure modes grows, which may restrict its applicability in large-scale problems without appropriate optimization. Additionally, determining suitable q-values and hesitant elements often requires expert intuition, which may introduce subjective bias. Future research may focus on improving the computational efficiency and developing automated mechanisms to better calibrate fuzzy parameters, making the method more robust and scalable. The method can be adapted and tested in different high-risk domains, such as healthcare, energy infrastructure, cybersecurity, and intelligent transportation systems, to evaluate its flexibility and domain-specific performance. Additionally, the fuzzy modelling environment can be enriched further by incorporating advanced fuzzy set extensions, such as spherical fuzzy sets, neutrosophic sets, or linear Diophantine fuzzy sets, to provide a more comprehensive framework for uncertainty representation. Lastly, future research may also consider hybridizing this approach with other robust MCDM techniques to evaluate its collaboration and consistency under different decision-making methods.

## Data Availability

The data are provided within the manuscript, and any requests regarding them should be directed to the corresponding author, Dr. Ali Köseoğlu.

## Abbreviations

AHP: Analytic hierarchy process
BWM: Best worst method
DM: Decision maker
EASA: European Union Aviation Safety Agency
FAA: Federal Aviation Administration
FM: Failure mode
FMEA: Failure modes and effects analysis
MABAC: Multi-attributive border approximation area comparison
MCDM: Multi-criteria decision-making
NIDM: Negative ideal decision matrix
PIDM: Positive ideal decision matrix
PROMETHEE: Preference ranking organization method for enrichment evaluations
q-ROHFS: q-Rung Orthopair Hesitant Fuzzy Set
RPN: Risk priority number
TOPSIS: Technique for order of preference by similarity to ideal solution
UAV: Unmanned aerial vehicle
VIKOR: VIsekriterijumska optimizacija i KOmpromisno Resenje
